# Biotransformation of *Momordica charantia* fresh juice by *Lactobacillus plantarum* BET003 and its putative anti-diabetic potential

**DOI:** 10.7717/peerj.1376

**Published:** 2015-10-29

**Authors:** Farhaneen Afzal Mazlan, M. Suffian M. Annuar, Yusrizam Sharifuddin

**Affiliations:** 1 Institute of Biological Sciences, University of Malaya, Kuala Lumpur, Malaysia; 2 Centre for Research in Biotechnology for Agriculture (CEBAR), University of Malaya, Kuala Lumpur, Malaysia

**Keywords:** *Momordica charantia*, *Lactobacillus plantarum*, Aglycones, Lactic acid fermentation, Biotechnology, Diabetes, α-glucosidase, Anti-diabetic

## Abstract

*Lactobacillus plantarum* BET003 isolated from *Momordica charantia* fruit was used to ferment its juice. *Momordica charantia* fresh juice was able to support good growth of the lactic acid bacterium. High growth rate and cell viability were obtained without further nutrient supplementation. In stirred tank reactor batch fermentation, agitation rate showed significant effect on specific growth rate of the bacterium in the fruit juice. After the fermentation, initially abundant momordicoside 23-O-*β*-Allopyranosyle-cucurbita-5,24-dien-7*α*,3*β*,22(*R*),23(*S*)-tetraol-3-O-*β*-allopyranoside was transformed into its corresponding aglycone in addition to the emergence of new metabolites. The fermented *M. charantia* juice consistently reduced glucose production by 27.2%, 14.5%, 17.1% and 19.2% at 15-minute intervals respectively, when compared against the negative control. This putative anti-diabetic activity can be attributed to the increase in availability and concentration of aglycones as well as other phenolic compounds resulting from degradation of glycosidic momordicoside. Biotransformation of *M. charantia* fruit juice via lactic acid bacterium fermentation reduced its bitterness, reduced its sugar content, produced aglycones and other metabolites as well as improved its inhibition of *α*-glucosidase activity compared with the fresh, non-fermented juice.

## Introduction

Non-dairy probiotic products are gaining importance due to increasing demand from consumers who suffer from lactose intolerance, allergic reaction to milk proteins and those who adopted vegetarian diet regime (Granato et al., 2010). Modification of fruit and vegetable matrices are possible today due to technological advances in food processing despite being relatively more complex than dairy products. Since most plants contain important nutrients such as sugars, minerals, vitamins, polyphenols, dietary fibers and antioxidants, they are ideal media for cultivation of probiotic culture which concomitantly enhance their nutritional functionality (Sheehan, Ross & Fitzgerald, 2007). More research is needed to explore fruit- and vegetable-based microbial growth media, as well as products and processes reengineering to supply consumers with new palatable and beneficial plant-based probiotic products.

*Momordica charantia* or bitter melon belongs to the Cucurbitaceae family; it is green in color and extremely bitter. It is reputed to possess anti-diabetic effects and has been widely used in traditional medicine for treating diabetic symptoms (Nhiem et al., 2010). *M. charantia* contains hypoglycaemic or insulin-like molecule, designated as ‘plant-insulin’, which has been found to be highly beneficial in lowering blood and urine sugar levels, hence it is potentially useful in managing Type II diabetes mellitus (T2DM) (Zaman, 1989). T2DM receives more attention than its Type 1 counterpart because it is regarded as a preventable disease. According to King and colleagues (1998), 500 million people worldwide will suffer from T2DM due to sedentary lifestyles, high calorie-nutrition and obesity between 2010 and 2025. Beside its anti-diabetic property, *M. charantia* is also reported to be anti-HIV, anti-tumor (Fang & Ng, 2011), an excellent source of phenolic compounds, antioxidants and anti-mutagen (Islam, Jalaluddin & Hettiarachchy, 2011). Currently available anti-diabetic medications such as acarbose can potentially cause gastrointestinal side effects such as bloating, abdominal discomfort, diarrhea and flatulence (Cheng & Fantus, 2005). Thus, natural inhibitors from dietary plants that possess anti-diabetic properties including inhibiting the activities of carbolytic enzymes such as *α*-glucosidase and *α*-amylase, with minimal side effects could be more acceptable alternatives to the commercial drugs, especially if they are incorporated into the daily diet.

In addition to producing probiotic culture in fruit and vegetable juices, research is also focusing on biotransformation of active compounds in these juices *via* fermentation with lactic acid bacteria (LAB) culture. LAB are used as starter culture in many fruit and vegetable fermentations due to their ability to produce *β*-glucosidase enzyme. This enzyme is an important catalyst in the liberation of aromatic compounds from glucoside precursors present in fruits and their fermentation products (Sestelo, Poza & Villa, 2004). Hence, new bioactive compounds with potential applications in health and medicine could be obtained from these biotransformation processes. In plants, aglycones such as terpenoids and isoflavones are known to be more potent than their glycosides. For example, aglycones of soy plants’ isoflavones were absorbed faster and available in higher amount in human plasma compared with their glucosides (Izumi et al., 2000). Fermentation of Canadian lowbush blueberry juice with the *Serratia vaccinii* improved its anti-diabetic activities compared with the unfermented juice (Vuong et al., 2007). Su and colleagues (2006) found that hydrolysis of ginsenoside Rg_3_ by *β*-glucosidase produced a very potent anti-tumor agent designated Rh_2_. Soyasapogenols B, the aglycones of soyasaponin were more cytotoxic toward cancer cells compared with their glycosides (Gurfinkel & Rao, 2003). Furthermore, isoflavone glycosides were found to require initial hydrolysis of the sugar moiety by *β*-glucosidase from the enteric bacteria prior to their absorption across the intestinal epithelium (Setchell et al., 2002). Hence, it is possible that biotransformation of *M. charantia* by *L. plantarum* in this study could potentially enhance its anti-diabetic activity compared with its fresh unfermented counterpart, apart from improvement of other properties pursued in this study.

Microbial biotransformation is widely used not only to improve medicinal and nutritional properties of fruit juices but also their taste characteristics. This include eliminating undesirable taste by modifying the juice’s phytochemical compositions (Hubert et al., 2008). *β*-glucosidase helps to improve the taste sensory properties of citrus fruit juices (Riou et al., 1998). Bitter compounds such as ginsenocides in ginseng (Kim et al., 2011), naringin in citrus fruit (Thomas, Smythe & Labbee, 2006), soyasaponins in soya bean (Yoshiki, Kudou & Okubo, 1998) and oleuropein in olive (Ciafardini et al., 1994) are present as glycosides. Microbial biotransformation such as LAB fermentation have been used in debittering and modifying the taste of fruit juices *via* hydrolysis of their glycosides (Marsilio, Lanza & Pozzi, 1996; Puri et al., 1996).

Similarly, lactic acid fermentation of *M. charantia* juice is hypothesized to reduce its bitterness and sugar content as well as increase its putative anti-diabetic activities due to the hydrolysis of glycosides by *β*-glucosidase from LAB. In this study, suitability of *M. charantia* juice for the growth of LAB was demonstrated. A wild lactic acid bacterium isolated from *M. charantia* itself was used as a starter culture. Fermentation conditions were optimized in a batch stirred tank reactor (STR) to achieve the highest growth rate. Anti-diabetic test was conducted on fresh and fermented juices to ascertain their anti-diabetic potentials via inhibition of *α*-glucosidase activity *in vitro*.

## Materials and Methods

### Raw material and chemicals

*Momordica charantia* of commercial maturity was purchased from a local supermarket (Kuala Lumpur, Malaysia). *β*-glucosidase enzymes, *p*-Nitrophenyl-*β*-D-glucopyranoside (*p*NPG) and reagents for the experiments were purchased from Sigma Laboratories (Sigma-Aldrich, St. Louis, MO), glucose oxidase reagent and glucose standard for the GM7 Multistat Analyser were purchased from Analox Instruments (London, UK). Other chemicals and solvents were purchased from Fisher Scientific (Leicestershire, UK).

### Isolation and physiological characterization of LAB

The process started with natural fermentation of fresh *M. charantia* fruits bearing wild lactic acid bacteria on their surfaces. The fruits (0.25 kg) were washed, sliced and mixed with freshly prepared sterilized solution of NaCl 6% (w/v), and then placed in 250 mL screw-capped glass vials. The salt provided hyper-osmotic environments to draw out juices and helped preserve the fruits while the fermentation progresses. The vials were incubated at 28 ± 1 °C for three days. LAB were isolated from the brines of naturally fermented *M. charantia* fruits after serial dilution of samples and plated on the Man, Rogosa and Sharpes (MRS) agar (Difco Laboratories, Detroit, MI). Agar plates were incubated at 30 °C for 48 hr. Fifty different colonies were randomly selected from the agar plates and transferred to new MRS agar plates. The isolates were purified by successive sub-culturing and screened using lactose utilization test as described by Thomas (1973). Lactose (1% w/v) as substrate and bromocresol purple (0.01% w/v) as acid indicator were included in the modified MRS agar. All 50 potential LAB isolates were streaked onto the modified MRS agar plates. Cell morphology of the selected strains was examined microscopically. Gram-positive and catalase–negative isolates were stored at −80 °C in separate glycerol stocks until further tests.

### Identification of LAB and growth profile

Four selected LAB isolates were further identified, by means of the API 50 CHL system (bioMerieux, Lyon, France) and 16S rRNA gene sequencing service. The growth profile for these selected LABs were determined and used to calculate the maximum growth rate (µ_max_) for each strain. LAB isolates were propagated in 10 mL of MRS broth and incubated at 30 °C for 12 hr at 180 rpm. Subsequently, the cultures were transferred into 90 mL shake flasks containing fresh MRS broth. At every 4-hr interval, 1 mL of culture was aliquoted into 1.5 mL centrifuge tube. Cells were harvested by centrifugation at 8,000-g for 5 min. The pellet was washed three times and re-suspended in sterile saline (0.9% w/v). The optical density (OD) at 600 nm was measured using spectrophotometer (Jasco V-630 Spectro, Japan). All fermentations were conducted in triplicates for 24 hr. Out of the four strains, an isolate designated as BET003 showed the highest specific growth rate.

### Sample preparation

Fresh *M. charantia* were thoroughly washed before being cut into small pieces. The seeds were removed and the fruit juice was obtained using a juice extractor (MJW171P, Panasonic). Then, the juice slurry was filtered through cheesecloths (2 kg fruits ≈ 1 L juice). Heat treatment was not implemented in the absence of bacterial growth following microbial enumeration on nutrient agar using samples from freshly prepared juice, which revealed no microbial growth after 48 hr at 30 °C.

### Inoculum and shake flask fermentation

The inoculum (*Lactobacillus plantarum* BET003) was prepared by streaking the culture from glycerol stock onto MRS agar at 30 °C for 48 hr. The starter culture was taken from a second subculture by transferring a single colony into 10 mL of MRS broth, which was subsequently incubated at 30 °C for 12 hr. Biomass was harvested by centrifugation at 8,000-g for 5 min and washed using sterile saline (0.9% w/v). Two hundred mL of *M. charantia* juice was dispensed into a shake flask. Then, a number of these flasks were inoculated with *L. plantarum* BET003 biomass suspended in 10 mL juice. This was followed by incubation at 30 °C for 24 hr at agitation rate of 180 rpm.

### Microbiological analysis

Viable cell counts (log CFU/mL) in the fermented *M. charantia* juice were enumerated by standard plate method with *Lactobacilli* MRS medium. Serial dilution of fermented juice was carried out using sterile saline solution (0.9% w/v). From these dilutions, 0.1 mL aliquots were plated in triplicate. The plates were incubated at 30 °C between 36 and 48 hr.

### pH, reducing sugar and viscosity measurements

pH changes were determined by measuring the pH of fermentation medium at regular intervals (pH meter-Eutech Instruments). Reducing sugar content was determined using dinitrosalicylic acid (DNS) method as described by Miller (1959). The viscosity of fermented *M. charantia* juice was measured using Vibro Viscometer SV–10 (Japan).

### Optimization of L. plantarum BET003 growth on M. charantia in batch stirred tank reactor (STR)

Optimization of fermentation conditions for bacterial growth was conducted in a 2 L stirred tank reactor (BIOSTAT^®^ A plus, Sartorius) at 1 L working volume of *M. charantia* juice. The optimum fermentation conditions were determined using central composite design (CCD) of two factors and two levels including five replicates at the center point. Two cultivation parameters *viz* agitation rate and initial inoculum volume (% v/v) were selected for optimization. Growth rate of *L. plantarum* BET003 was the main response and determined based on viable cell counts. Agitation speed was studied from 200 to 400 rpm. Initial inoculum volume was varied at 1%, 5% and 10% (v/v) based on the recommendation for probiotic foods with minimal counts of 7.00 Log CFU/mL. Fresh juice medium was inoculated with a 12-hr old culture prepared as previously described. Cultivation temperature was set at 30 °C. Samples were taken at every 4 hr during the 24 hr fermentation period to determine viable cell count and reducing sugar concentration. pH and dissolved oxygen partial pressure (*p*O_2_) real time readings were obtained from online sensors’ measurements. At optimum condition, samples were analysed for *β*-glucosidase activity at every 2 hr interval for 24 hr to determine the time of highest enzyme activities.

The STR was equipped with an automated controller for temperature and agitation speed. Rushton turbine impeller and four baffles were installed. The reactor was connected to thermostated waterbath (WiseCircu) that helped regulate the temperature *via* cooling finger and heating pad. The fermentation was carried out as micro-aerobic system since gas sparging was not provided. The *p*O_2_ showed 0% reading as the number of viable cells increased.

### Determination of *β*-glucosidase activity during fermentation in STR

The *β*-glucosidase activity was monitored throughout the batch fermentation. Enzyme activity assay was performed at every 2 hr interval for 24 hr. A half mL cell free extracts of fermented juice was added to 0.5 mL of 5 mM *p*-Nitrophenyl-*β*-D-glucopyranoside (*p*NPG) and 2 mL of sodium phosphate buffer 50 mM, pH 6. Then, the mixture was incubated at 40 °C for 15 min. The reaction was stopped by adding 1.0 mL of 1 M Na_2_CO_3_. The released *p*-nitrophenol (*p*NP) was measured at 410 nm using spectrophotometer (Jasco V-630 Spectro, Japan). One unit of enzyme activity was defined as the amount that released 1 µmol *p*NP per min.

### LC/MS/MS analysis

Analysis of triterpenes present in the juice was carried out using LC/MS/MS. The ionization mode was set into negative mode. Sample extracts were filtered with nylon filter (0.2 µm pore size). Twenty µL of sample was injected into Phenomenex Aqua C18–50 mm × 2.0 mm × 5 µM column equipped with AB SCIEX 3,200 QTrap MS/MS system coupled with Perkin Elmer FX15 UHPLC. The mobile phase was water with 0.1% formic acid (v/v) and 5 mM ammonium formate (Solvent A) and acetonitrile with 0.1% formic acid and 5 mM ammonium formate (Solvent B). The gradient program were as follows: 10% to 90% solvent B from 0.01 min to 8 min hold for 2 min and back to 10% solvent B in 0.1 min and re-equilibrated for 5 min with total running time of 15 min. In this study, LC/MS/MS analyses of phytochemical profiles were carried out for fermentation time of 6-, 12-, and 24-hr respectively. Fresh *M. charantia* juice was used as control.

### Anti-diabetic test (*α*-glucosidase inhibition *in vitro*)

The *α*-glucosidase inhibitory potential of fresh and fermented *M. charantia* juices was assessed by measuring glucose production inhibition using maltose as substrate, as described by Iwai (2008) with modifications. The fermented juice samples examined in the *α*-glucosidase inhibition assay were those which underwent successful fermentation by *L. plantarum* BET003. New stocks of fresh and fermented *M. charantia* juices without *L. plantarum* BET003 presence were used for every independent experiments conducted. Rat small intestinal acetone powder was homogenized in 56 mM maleate buffer (pH 6.0) in a 1:9 ratio (w/v) and the mixture was rigorously stirred at room temperature for 40 min. Subsequently, the mixture was centrifuged at 1,500-g for 5 min. The supernatant was collected as crude enzyme solution to be used in *α*-glucosidase inhibitory assay. Maltose as substrate was prepared in phosphate-buffered saline (PBS; pH 7.4) at 10 mg/mL. Independent samples of fresh and fermented *M. charantia* juices were freshly prepared and 150 µL of the juice was mixed with 300 µL of maltose and 10 µL of the enzyme supernatant. The reaction mixture was gently vortexed using a Whirlimixer for 1 min at room temperature and incubated at 37 °C in a waterbath with agitation for 60 min. The concentrations of glucose produced per sample were measured in duplicates at every 15 min intervals for 60 min by transferring 10 µL of the reaction mixture into a GM7 Multistat Analyser (Analox Instruments, UK). The concentrations of glucose produced from maltose in the presence of fresh and fermented juices were compared using paired sample *t*-test. The negative control was PBS in the reaction mixture containing maltose and rat intestinal enzyme supernatant. Acarbose (1 mg/mL) was added to the mixture instead of PBS and served as the positive control. The experiments were independently repeated for four times using freshly prepared samples and *p* < 0.05 was considered statistically significant.

### Calculation

Specific growth rate *μ* (h^−1^), was calculated using

(1) \begin{eqnarray*} \mu =\frac{\ln {X}_{2}-\ln {X}_{1}}{{t}_{2}-{t}_{1}}. \end{eqnarray*}

### Statistical analyses

Analysis of variance (ANOVA) and surface regression were carried out to determine the statistical significance of model terms and fit a regression relationship relating the experimental data to independent variables, respectively. The experimental design matrix, data analysis, and optimization procedures were carried out using DOE software (Design-Expert software from Stat-Ease Inc.). Central Composite Design was used to create a process map allowing the determination of significant individual factors as well as finding theoretical optimal condition.

## Results and Discussion

### Isolation and characterization of lactic acid bacteria

Natural fermentation of fresh, sliced fruits of *M. charantia* in brine was carried out to encourage the proliferation of lactic acid bacteria (LAB). MRS agar added with lactose and bromocresol purple as pH indicator was used to isolate lactic acid bacteria from the mixed microbial population. Only four strains out of 50 plates showed positive results in lactose utilization test. A 12 hr sample for each isolate was also observed under a light microscope. All four provisional LAB strains showed rod-shape and purple color after Gram staining.

#### API test of provisional LAB strains

Four LAB strains designated as BET012, BET003, BET022 and BET028 were identified to be *Lactobacillus plantarum* (99.9%), (77.5%), (99.9%) and (99.9%) respectively, based on biochemical assay using API 50 CHL kit (bioMérieux Inc., Durham, NC, USA).

#### Growth profiles

**Figure 1:**
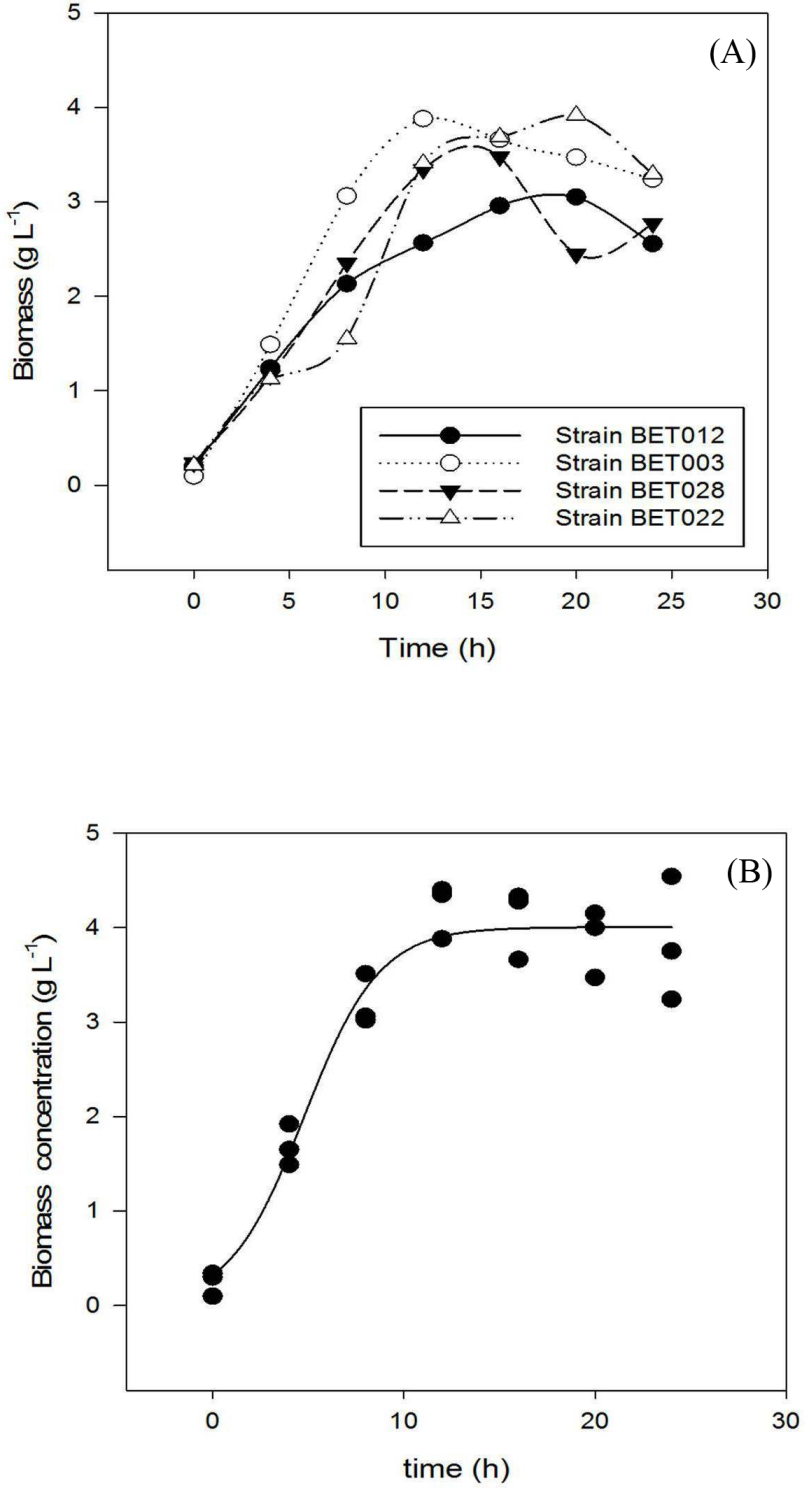
Growth profiles during 24 h fermentation of MRS medium at 30 °C: (A) LAB strains BET012, BET003, BET028, BET022; (B) BET003 in triplicate.

Growth profiles of the four LAB isolates are shown in Fig. 1. Specific growth rates of BET012, BET003, BET022 and BET028 were calculated at 0.18, 0.21, 0.17 and 0.19 h^−1^ respectively. The isolate designated as *L. plantarum* BET003 showed the highest specific growth rate on MRS medium among the four isolates examined. Hence, *L. plantarum* BET003 was chosen for the fermentation of *M. charantia* fresh juice due to its fast growth rate. In addition, this strain was further subjected to 16S ribosomal RNA sequencing service to determine the genetic lineage.

#### 16S ribosomal RNA sequencing and phylogenetic tree

**Figure 2:**
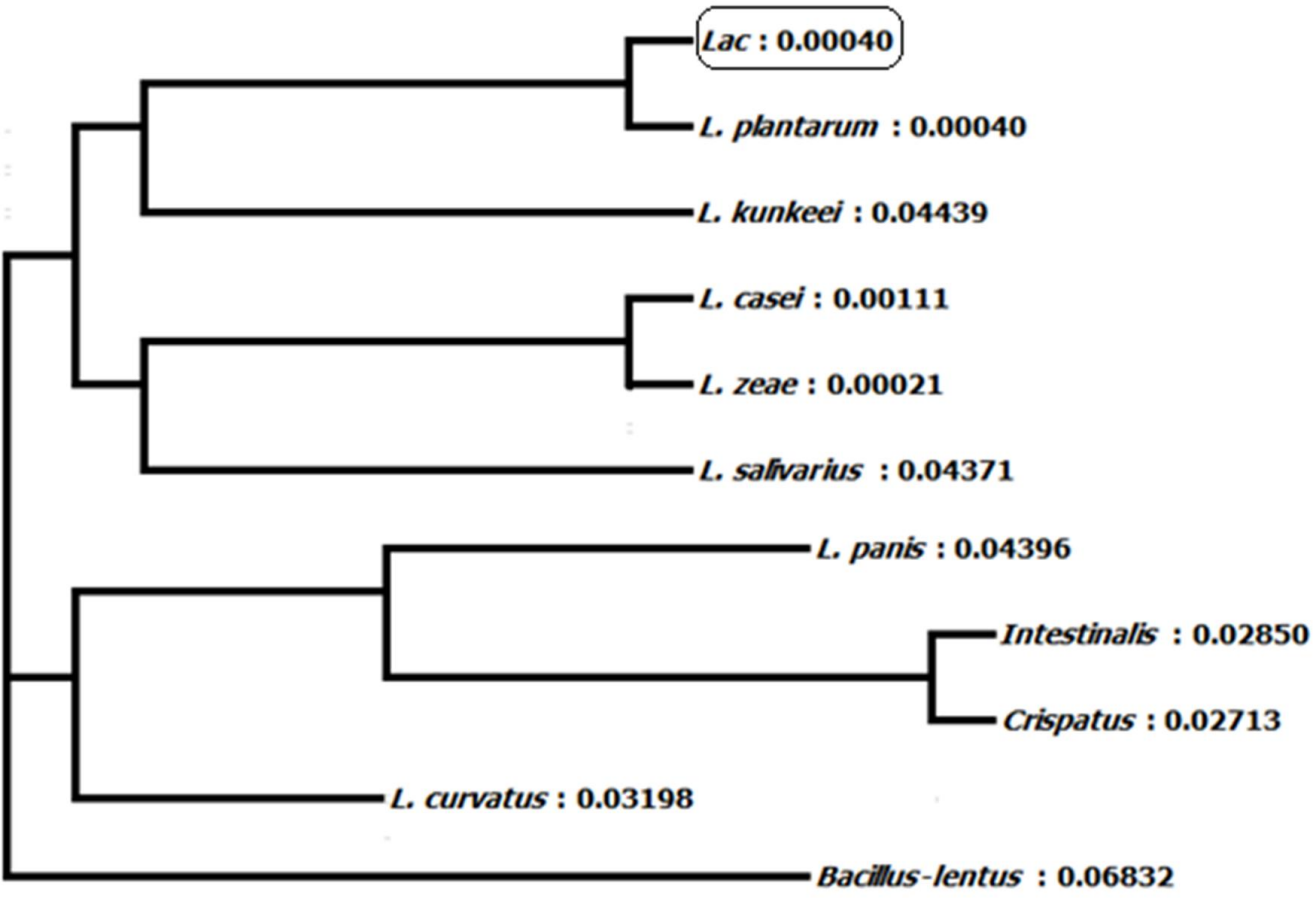
Phylogeny tree of isolated lactic acid bacteria ‘Lac’ (i.e., BET003).

Homology of 16S ribosomal RNA sequences of *L. plantarum* BET003 strain was compared with those in the GenBank of National Center for Biotechnology Information (NCBI, Bethesda, MD, USA). The lineage of strain BET003 is in the root of domain bacteria, in phylum of Firmicutes, class of Bacilli, order of *Lactobacillales*, family of *Lactobacillaceae* and genus of *Lactobacillus*. As shown in Fig. 2, strain BET003 is closely related to *Lactobacillus plantarum* based on the 16S rRNA gene sequence similarity search and phylogenetic analysis.

### Shake flask fermentation of *M. charantia* juice with *L. plantarum* BET003

#### Microbiological analysis

**Figure 3:**
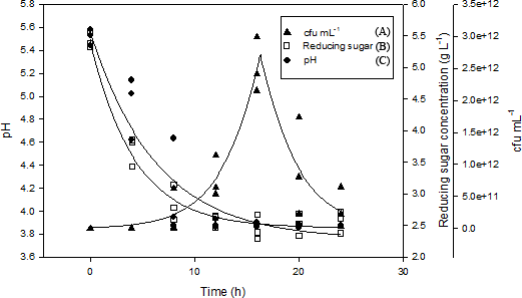
Shake flask fermentation of *M. charantia* juice with *L. plantarum* BET003 at 30 °C for 24 h: (A) cell viability (Log CFU mL^−1^); (B) reducing sugar; (C) pH.

Figure 3 showed the changes in viable cell numbers of the *L. plantarum* BET003 in shake flasks fermentation of *M. charantia* fresh juice at 30 °C for 24 hr with an initial cell number of 10^8^ CFU/mL. From the results, the growth of *L. plantarum* BET003 was slow during the early hours of fermentation. After 5 hr of fermentation, the culture exhibited accelerated growth. The total plate count increased exponentially from 10^8^ to 10^12^ CFU/mL until 16 hr of fermentation followed by slight viability loss between 16 and 24 hr, where the cell viability decreased to 10^11^ CFU/mL. The results showed that the fast growth of *L. plantarum* BET003 required a short batch cultivation period. The ability of the cells to rapidly utilize fermentable sugars in the juice for growth without further nutrient supplementation proved the suitability of *M. charantia* fresh juice as a growth medium for *L. plantarum* BET003.

The reducing sugar concentration rapidly decreased from 5.5 to 3.0 g/L within 8 hr of fermentation period as shown in Fig. 3. Between 8 and 12 hr of fermentation, the rate of sugar utilization slowed down and its concentration decreased slightly to 2.5 g/L. Between 12 and 24 hr fermentation, no further changes in reducing sugar concentration was observed. Rate of sugar utilization was rapid when *L. plantarum* BET003 was in its exponential growth phase. However, since there was approximately 2.5 g/L reducing sugar remaining in the juice medium after the fermentation, this indicated that the carbon source was not a limiting factor for bacterial growth. Instead, pH value appears to be the limiting factor that inhibited further growth of *L. plantarum* BET003. The reduction of sugar content in the *M. charantia* fermented juice is a desirable characteristic in making the juice more acceptable for diabetic consumption.

Rapid decrease in pH was also observed during the first 16 hr of fermentation period where pH decreased from 5.50 to approximately 3.85 (Fig. 3). No further pH changes were observed from 16 to 24 hr fermentation period. The decrease in pH was concomitant to the increase in viable cell count during the first 16 hr of fermentation. *L. plantarum, L. acidophilus* and *L. casei* have been reported to grow well in fruit matrices due to their excellent tolerance of acidic environments compared with other LABs (Yoon, Woodams & Hang, 2006). A rapid decrease of pH during the early stage of fermentation is an important indicator of end product quality. The increase in acidity significantly minimizes the activities of spoilage bacteria and contributes to the pleasant taste and desirable aroma (Karovičová & Kohajdová, 2003). However, such acidity increase should not exceed below pH 3.6 as it is undesirable from the sensory perception aspect.

From the results obtained in this study, the total cell viability for *L. plantarum* BET003 was maintained at relatively high number (∼10^11^ CFU/mL) throughout the fermentation process.

In terms of liquid flow behavior, viscosity of fermented *M. charantia* juice was measured at 2.89 mPa s compared with the viscosity of commercial yogurt drink at 4.80 mPa s. Thus, the fermented juice exhibited relatively easy flow behavior compared with dairy-based probiotic beverage, which may expedites downstream processing later.

### Optimization of batch fermentation in STR

In Table 1, specific growth rate as a function of agitation rate and initial inoculum volume variables was presented. Specific growth rate ranging from 0.24 to 0.64 h^−1^ was observed. For all the initial inoculum volume studied, there was a general increase in specific growth rate of *L. plantarum* BET003 with higher agitation rate (Table 1). Linear, quadratic and interaction effects of agitation rate (*x*_1_) and initial inoculum volume (*x*_2_) on specific growth rate (*Y*) were also investigated. The results indicated that the response surface model fitted was significant (*p* < 0.05) and explanatory for both response variables studied. High coefficient of correlation (*R*^2^ > 0.7974) was observed for all polynomial regression models tested. Hence, more than 80% of the response variation could be accurately explained as a function of the two independent parameters. The model lack of fit was not significant relative to the pure error (*p* > 0.05).

**Table 1:** Specific growth rate (h^−1^) of *L. plantarum* BET003 in stirred tank reactor as a function of agitation rate and initial inoculum volume (□ 8% maximum).

	Inoculum volume (% v/v)		
Agitation rate (rpm)	1	5	10
200	0.24	0.36	0.30
300	0.57	0.59	0.58
400	0.64	0.55	0.59

**Figure 4:**
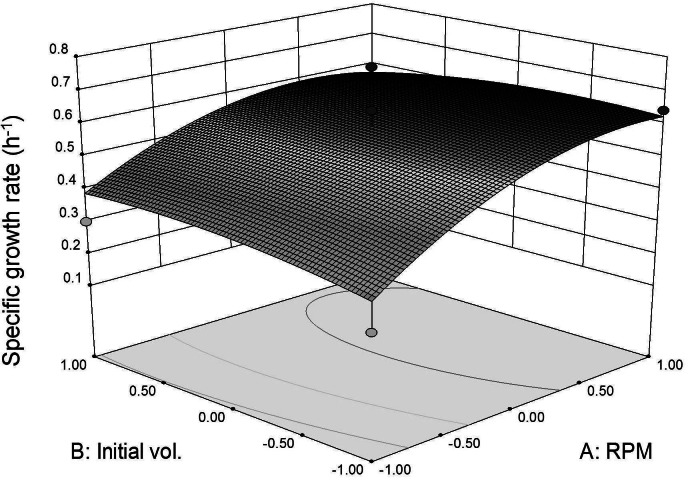
Specific growth rate as function of agitation speed and inoculum initial volume (30 °C for 24 h).

Three dimensional response surface plot was constructed to visualize the interaction effects between coded agitation rate and initial inoculum volume variables on *L. plantarum* BET003’s specific growth rate at 30 °C (Fig. 4). As shown in the response surface plot and ANOVA, the agitation speed exhibited significant effect (*p* < 0.05) for the main-, quadratic- and interaction effects towards the specific growth rate. The results indicated that faster agitation rate increased the growth rate due to better mass transfer within the culture medium. However, based on the response surface model and ANOVA, the main-, quadratic- and interaction effects of initial inoculum volume were not significant (*p* > 0.05) on specific growth rate. The results indicated that the specific growth rate was not dependent on the initial volume of inoculum. Instead, initial inoculum volume affects fermentation time taken to reach maximum growth. Initial inoculum volume of 10% (v/v) reached maximum growth at 8 h whereas for the 5% (v/v) and 1% (v/v) initial inoculum volumes, both showed maximum growth at 12 and 16 h respectively. Thus, increasing initial volume of inoculum to 10% (v/v) resulted in shorter fermentation period due to substantially higher initial cell concentration. The optimum fermentation condition for highest specific growth rate response was at 400 rpm agitation rate and 10% (v/v) initial volume of inoculum. The same conditions were applied to obtain fermented juice samples for the evaluation of *β*-glucosidase activities and anti-diabetic activity (*α*-glucosidase inhibitory potential).

### *β*-glucosidase assay

**Figure 5:**
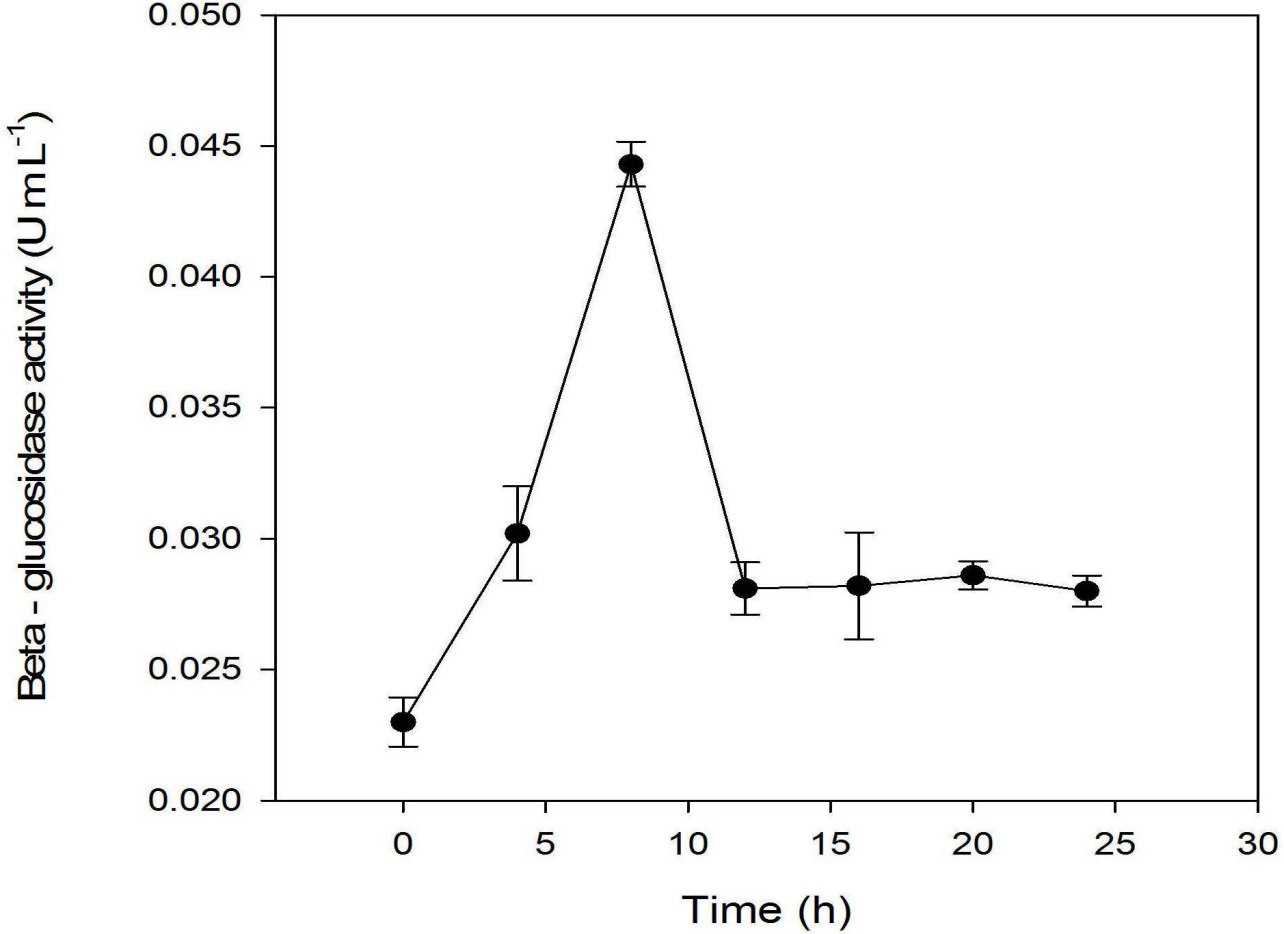
Assay of *β*-glucosidase activity: fermentation with 10% starter cultures *L. plantarum* BET003 at 30 °C and 400 rpm of agitation speed.

Other previous studies showed that *β*-glucosidase activities in microorganisms are highly correlated to the hydrolysis of glucoside into their corresponding aglycones (Chien, Huang & Chou, 2006; Pyo, Lee & Lee, 2005). Thus, *β*-glucosidase activities as a function of fermentation time were analyzed (Fig. 5). The profile showed that the *β*-glucosidase activities were correlated with the growth of *L. plantarum* BET003. At 8 hr after inoculation, the enzyme activities increased to a maximum ∼(5.8 U/mL) and precipitously decreased after 8 hr. It became constant thereafter until the termination of fermentation.

### LC/MS/MS analysis

**Figure 6:**
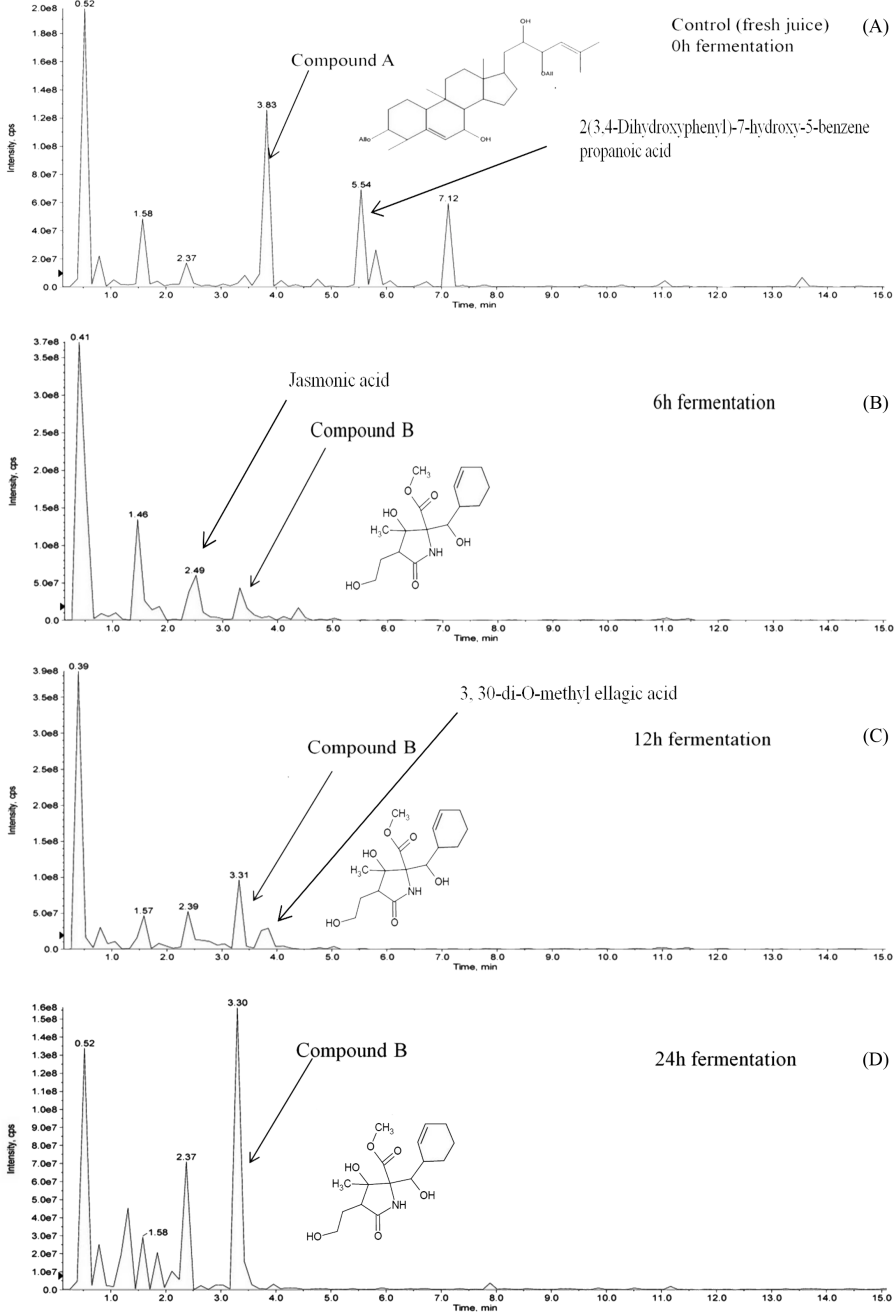
LCMS/MS analysis of *M. charantia* juice: (A) fresh juice as control; and fermented juice for (B) 6 h; (C) 12 h; (D) 24 h.

Hydrolysis of momordicoside during fermentation of *L. plantarum* BET003 culture in *M. charantia* fresh juice was investigated. After 24 hr fermentation under optimum condition, initially abundant momordicoside identified as 23-*O*-*β*-Allopyranosyle-cucurbita-5,24-dien-7*α*,3*β*,22(*R*),23(*S*)-tetraol-3-*O*-*β*-allopyranoside (compound A, 2.1 ± 0.2 mg/mL) was totally absent (Fig. 6A). The concentration of its corresponding aglycones was increased and there were other new metabolites produced *via* biotransformation during the fermentation process. In this study, a metabolite known as methyl 2-[cyclohex-2-en-1-yl(hydroxy)methyl]-3-hydroxy-4-(2-hydroxyethyl)-3-methyl-5-oxoprolinate (compound B) was significantly produced at the end of fermentation ∼2.5 ± 0.2 mg/mL (Figs. 6B–6D). The results suggested that stoichiometric conversion of compound A to compound B and other phenolic compounds over the course of the whole fermentation period may have occurred. Compound A was hypothesized to be converted to compound B *via* enzyme hydrolysis from *L. plantarum* BET003 which involves a two-step process. The first step was attributed to glucosidase-like enzymatic hydrolysis of the glycosidic linkage to release the aglycone. After the glycosidic linkage was completely hydrolyzed, compound B and other phenolic compounds materialized. The second step could have involved esterase activities that hydrolyzes the ester to acid and alcohol (Marsilio, Lanza & Pozzi, 1996). While the two-step enzymatic hydrolysis hypothesis is plausible, further studies need to be carried out in order to confirm the conversion route. Fermentation of *M. charantia* juice also produced other phenolic compounds such as ellagic acid as other major products. Ellagic acid was observed at 12 and 24 hr fermentation time. Jasmonic acid was detected after 6 hr but disappeared at 12 hr fermentation time until the end. Biotransformation of other saponin-containing plant such as ginseng and soya bean with lactic acid bacteria that modified their phytochemical profiles resulting in improved bioactive properties have been reported (Bae et al., 2004; Gurfinkel & Rao, 2003). Rg3, a percusor of Rh2 ginsenoside, showed a potent antitumor properties but due to its extremely low concentration in normal ginseng, Rg3 was produced *via* biotransformation by lactic acid fermentation (Cheng et al., 2008). The aglycone of soyasaponin called soyasapogenol, was more cytotoxic toward cancer cells compared with its glycosides form (Gurfinkel & Rao, 2003). Biotransformation of soyasaponin by *Lactobacillus rhamnosus* has been reported to occur at specific time points and resulted in different phytochemical profiles (Zhang et al., 2012). Thus, by controlling the fermentation time, specific phytochemical profile may be obtained and desirable products could be enhanced through optimization.

### Anti-diabetic assay (*α*-glucosidase inhibition *in vitro*)

**Figure 7:**
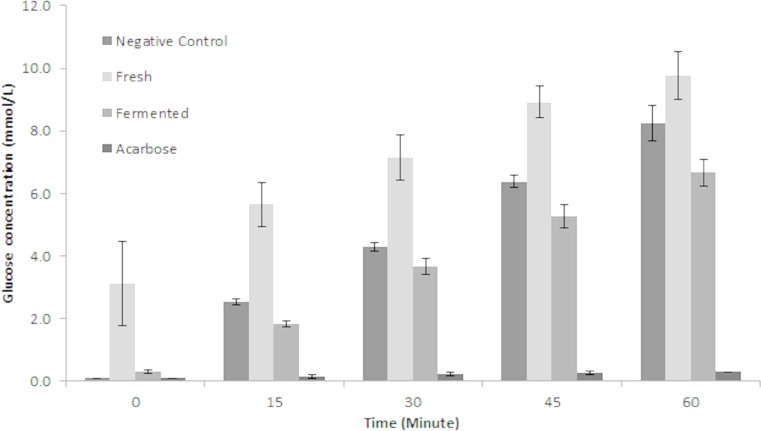
Comparison of anti-*α* glucosidase inhibitory activity between fresh and fermented *M. charantia* juices measured as glucose production (mmol/L) at every 15-minute intervals for 60 min. Acarbose (1 mg/mL) was used as the positive control. The bar representing mean of four independent experiments with error bars of standard deviation.

To the best of our knowledge, this is the first report on putative *α*-glucosidase inhibitory activity by fermented *M. charantia* juice. There are many strategies in managing hyperglycaemia and interrupting digestion of dietary carbohydrate by inhibiting the activities of carbolytic enzymes such as *α*-glucosidase is one popular strategy. Currently, there are several efficient anti-diabetic drugs namely voglibose and acarbose which inhibits *α*-glucosidase activity but the continuous use of these drugs is often linked to undesirable side effects such as flatulence and other adverse gastrointestinal discomforts (Van de Laar, 2008). Therefore, the prospect of retarding glucose production *via* dietary means is very attractive. As shown in Fig. 7, fermented *M. charantia* juice exhibited significant *α*-glucosidase inhibitory activity as measured by the concentrations of glucose produced at every 15 min intervals during 60 min incubation period compared with both fresh *M. charantia* juice and negative control. The fermented juice consistently reduced glucose production from maltose by 27.2%, 14.5%, 17.1% and 19.2% at every 15-minute intervals respectively, when compared against the negative control. The fresh *M. charantia* juice did not inhibit *α*-glucosidase activity *in vitro* as measured by glucose production in this study. Furthermore, the sugar content of fresh *M. charantia* juice was higher than in the fermented juice, in addition to continuous production of additional glucose during the anti-diabetic assay. In contrast, LAB steadily utilized reducing sugars during the fermentation period and significantly decreased the sugar content in the fermented juice after fermentation. The decreased sugar content and inhibition of *α*-glucosidase activity properties exhibited by fermented *M. charantia* juice could be beneficial for consumption by diabetics, subject to further studies.

It is hypothesized that the putative anti-diabetic property of fermented *M. charantia* juice in inhibiting glucose production compared with its fresh counterpart was due to the increase in concentration and availability of aglycones, as well as other phenolic compounds resulting from degradation of glycosidic momordicoside *via* biotransformation process mediated by *L. plantarum* BET003. During the fermentation process, enzymes from the lactic acid bacterium modified the phytochemical profile and thus altered the biological activities of momordicoside. Hence, the increased presence of compound B (methyl 2-[cyclohex-2-en-1-yl(hydroxy)methyl]-3-hydroxy-4-(2-hydroxyethyl)-3-methyl-5-oxoprolinate) after 8 hr fermentation of *M. charantia* juice with *L. plantarum* BET003 can be correlated to the increased *α*-glucosidase activity *in vitro* compared with both the fresh juice and negative control. Furthermore, an alteration in *M. charantia* saponin composition is likely to influence its biological activities. Aglycones were reported to be more efficiently absorbed in human subjects and available at higher concentrations in plasma compared with their glucosides (Izumi et al., 2000). Thus, biotransformation of fermented *M. charantia* juice using *L. plantarum* BET003 was demonstrated to increase the bioactive property of *M. charantia* in hyperglycemia management *via* anti-*α* glucosidase inhibition and may be useful in reducing the risk of Type II diabetes. Further studies on the *α*-glucosidase inhibitory potential of fermented *M. charantia* juice is currently being pursued to elucidate the bioactive compound(s) involved and determine their dose–response relationships.

In conclusion, we isolated wild *Lactobacillus plantarum* BET003 from *Momordica charantia* which was utilized for fermentation of its fruit juice. The fresh juice provided good growth environment for the LAB culture without further nutrient supplementation and under optimum fermentation conditions, high cell viability and specific growth rate were successfully achieved. It was also demonstrated that biotransformation of *M. charantia* fruit juice *via* lactic acid bacterium fermentation reduced its bitterness, reduced its sugar content, produced aglycones and other metabolites as well as improved its putative anti-diabetic *α*-glucosidase inhibitory potential compared with the fresh unfermented juice.

## Supplemental Information

10.7717/peerj.1376/supp-1 Supplemental Information 1 Bacteria identification by 16S rRNA gene sequencing

10.7717/peerj.1376/supp-2 Supplemental Information 2 Compilation of fermentation data

10.7717/peerj.1376/supp-3 Supplemental Information 3 ANOVA for optimization of fermentation

10.7717/peerj.1376/supp-4 Supplemental Information 4 Detection of ellagic acid as a fermentation product

10.7717/peerj.1376/supp-5 Supplemental Information 5 Detection of jasmonic acid as a fermentation product

10.7717/peerj.1376/supp-6 Supplemental Information 6 Comparison of anti-*α* glucosidase inhibitory activity between fresh and fermented *M. charantia* juices measured as glucose production (mmol/L) at every 15-minute intervals for 60 min Acarbose (1 mg/mL) was used as the positive control.
