# Supplementary material for: Biotransformation of Momordica charantia fresh juice by Lactobacillus plantarum BET003 and its putative anti-diabetic potential
Source: PeerJ. 2015 Oct 29;3:e1376. doi: 10.7717/peerj.1376 (PMC4631465; doi:10.7717/peerj.1376)
Supplement: Supplemental Information 1 [file peerj-03-1376-s001.pdf]

All Special Shop Trading (SA-0148761-U)
No. 51, Jalan SP 6/3, Bandar Saujana Putra
42610 Lebuhraya ELITE, Selangor Darul Ehsan, MALAYSIA
Tel/Fax: +603 7726 2810 Email: asst2010@yahoo.com

Service Report

Bacterial Identification By 16S rRNA Gene Sequencing

Date: 01 April 2012

No.	Sample ID	Comment	Remarks
01.	Lac	Bacterium "Lac" is closely related to ***Lactobacillus plantarum*** based on the 16S rRNA gene sequence similarity search and phylogenetic analysis.	Supplied data:<br>1. DNA Sequencing results<br>2. Assembled 16S rRNA gene sequence<br>3. Top 10 reported hits to the query<br>4. Multiple sequence alignment of 10 orthologous sequences and an outgroup<br>5. Phylogenetic tree

Prepared by
David Lee

*The chromatogram is saved as *ab1* files. The recommended trace viewer is Sequence Scanner 1.0 from http://www.appliedbiosystems.com/sequencescanner

Assembled 16S rRNA Gene Sequence

>Lac
GCCTAATACATGCAAGTCGAACGAACTCTGGTATTGATTGGTGCTTGCATCATGATTTACATTTGAGTGAGTGGCGAA
CTGGTGAGTAACACGTGGGAAACCTGCCCAGAAGCGGGGGATAACACCTGGAAACAGATGCTAATACCGCATAACAA
CTTGGACCGCATGGTCCGAGTTTGAAAGATGGCTTCGGCTATCACTTTTGGATGGTCCCGCGGCGTATTAGCTAGATG
GTGGGGTAACGGCTCACCATGGCAATGATACGTAGCCGACCTGAGAGGGTAATCGGCCACATTGGGACTGAGACACG
GCCCAAACTCCTACGGGAGGCAGCAGTAGGGAATCTTCCACAATGGACGAAAGTCTGATGGAGCAACGCCGCGTGAG
TGAAGAAGGGTTTCGGCTCGTAAAACTCTGTTGTTAAAGAAGAACATATCTGAGAGTAACTGTTCAGGTATTGACGGT
ATTTAACCAGAAAGCCACGGCTAACTACGTGCCAGCAGCCGCGGTAATACGTAGGTGGCAAGCGTTGTCCGGATTTAT
TGGGCGTAAAGCGAGCGCAGGCGGTTTTTTAAGTCTGATGTGAAAGCCTTCGGCTCAACCGAAGAAGTGCATCGGAA
ACTGGGAAACTTGAGTGCAGAAGAGGACAGTGGAACTCCATGTGTAGCGGTGAAATGCGTAGATATATGGAAGAAC
ACCAGTGGCGAAGGCGGCTGTCTGGTCTGTAACTGACGCTGAGGCTCGAAAGTATGGGTAGCAAACAGGATTAGATA
CCCTGGTAGTCCATACCGTAAACGATGAATGCTAAGTGTTGGAGGGTTTCCGCCCTTCAGTGCTGCAGCTAACGCATT
AAGCATTCCGCCTGGGGAGTACGGCCGCAAGGCTGAAACTCAAAGGAATTGACGGGGGCCCGCACAAGCGGTGGAG
CATGTGGTTTAATTCGAAGCTACGCGAAGAACCTTACCAGGTCTTGACATACTATGCAAATCTAAGAGATTAGACGTTC
CCTTCGGGGACATGGATACAGGTGGTGCATGGTTGTCGTCAGCTCGTGTCGTGAGATGTTGGGTTAAGTCCCGCAAC
GAGCGCAACCCTTATTATCAGTTGCCAGCATTAAGTTGGGCACTCTGGTGAGACTGCCGGTGACAAACCGGAGGAAG
GTGGGGATGACGTCAAATCATCATGCCCCTTATGACCTGGGCTACACACGTGCTACAATGGATGGTACAACGAGTTGC
GAACTCGCGAGAGTAAGCTAATCTCTTAAAGCCATTCTCAGTTCGGATTGTAGGCTGCAACTCGCCTACATGAAGTCG
GAATCGCTAGTAATCGCGGATCAGCATGCCGCGGTGAATACGTTCCCGGGCCTTGTACACACCGCCCGTCACACCATG
AGAGTTTGTAACACCCAAAGTCGGTGGGGTAACCTTTTAGGAACCAGCCGCCTAAG

The top 10 reported hits to the query 16S rRNA gene sequence

Accession	Description	Score	E Value
JQ340030.1	Lactobacillus plantarum strain R17 16S ribosomal RNA gene, partial sequence	2678 bits	0.0
AB690244.1	Lactobacillus plantarum gene for 16S rRNA, partial sequence, strain: JCM 1100	2678 bits	0.0
AL935263.2	Lactobacillus plantarum WCFS1 complete genome	2678 bits	0.0
FR775893.1	Lactobacillus plantarum subsp. plantarum partial 16S rRNA gene, type strain CIP 103151T	2678 bits	0.0
FR871789.1	Lactobacillus pentosus MP-10 draft genome, annotated contig00046	2678 bits	0.0
HQ384301.1	Lactobacillus pentosus strain 5-36 16S ribosomal RNA gene, partial sequence	2678 bits	0.0
GU552552.1	Lactobacillus plantarum strain KW30 16S ribosomal RNA gene, complete sequence; and 16S-23S ribosomal RNA intergenic spacer, partial sequence	2678 bits	0.0
AB603688.1	Lactobacillus plantarum subsp. plantarum gene for 16S rRNA, partial sequence, strain: UB9	2678 bits	0.0
AB603687.1	Lactobacillus plantarum subsp. plantarum gene for 16S rRNA, partial sequence, strain: UB8	2678 bits	0.0
AB603686.1	Lactobacillus plantarum subsp. plantarum gene for 16S rRNA, partial sequence, strain: UA6	2678 bits	0.0

Multiple Sequence Alignment of 10 Orthologous Sequences and an Outgroup

```
CLUSTAL 2.1 multiple sequence alignment

Lac                  ---------------------------------------GCCTAATACATGCAAGTCGAA 21
L.plantarum          --------------------------ACGCTGGCGGCGTGCCTAATACATGCAAGTCGAA 34
L.panis              -------------------------------------GTGCCTAATACATGCAAGTCGAG 23
L.curvatus           ----------------------ACGAACGCTGGCGGCGTGCCTAATACATGCAAGTCGAA 38
L.intestinalis       --------------------GGACGAACGCTGGCGGCGTGCCTAATACATGCAAGTCGAG 40
L.crispatus          GAGAGTTTGATCCTGGCTCAGGACGAACGCTGGCGGCGTGCCTAATACATGCAAGTCGAG 60
L.casei              ---------------------GATGAACGCTGGCGGCGTGCCTAATACATGCAAGTCGAA 39
L.kunkeei            ---------------------GACGAACGCTGGCGGCGTGCCTAATACATGCAAGTCGAA 39
L.zeae               ---------------------GATGAACGCTGGCGGCGTGCCTAATACATGCAAGTCGAA 39
L.salivarius         ---------------------GACGAACGCTGGCGGCGTGCCTAATACATGCAAGTCGAA 39
Bacillus-lentus      -AGAGTTTGATCCTGGCTCAGGACGAACGCTGGCGGCGTGCCTAATACATGCAAGTCGAG 59
                                                            ********************

Lac                  CGAA-CTCTGGTA--TTGATTG-G--TGCTTGCATCA-TGAT----TTAC--ATTTG-AG 67
L.plantarum          CGAA-CTCTGGTA--TTGATTG-G--TGCTTGCATCA-TGAT----TTAC--ATTTG-AG 80
L.panis              CGCA-CTGGCCCAA-CTGATATGACGTGCTTGCACT---GATTTGACGATGGATTACCAG 78
L.curvatus           CGCA-CT---CTCGTTAGATTGAAGAAGCTTGCTTCT--GATT-GATAACATTTGA---G 88
L.intestinalis       CGAG-CTGAACCAG-CAGATTCA-----CTTCGGT----GAT--GACGCTGGGA-AC--G 84
L.crispatus          CGAG-CTTGCCTAGATGAATTTGG--TGCTTGCACCA--GAT--GAAACTAGAT-ACAAG 112
L.casei              CGAG-TT---TTGG-TCGATGAACGGTGCTTGCACTG-WGAT-------TCRACTTAAAA 86
L.kunkeei            CGAG-CTCTCCCAAATTGATTTTA--TGCTTGCATAAATGAT----TTTTGGATTCGGAG 92
L.zeae               CGAG-TT---TTGG-TCGATGAACGGTGCTTGCATCG-TGAT-------TCAACTTAAAA 86
L.salivarius         CGAAACTTTCTTACACCGA------ATGCTTGCATTC---ACC---------GTAAGAAG 81
Bacillus-lentus      CGAA-----------TGGATGGGA---GCTTGCTCCC-----------------AGAAG 87
                     **                *         ***

Lac                  T-GAGTGGCGAACTGGTGAGTAACACGTGGGAAACCTGCCCAGAAGCGGGGGATAACACC 126
L.plantarum          T-GAGTGGCGAACTGGTGAGTAACACGTGGGAAACCTGCCCAGAAGCGGGGGATAACACC 139
L.panis              T-GAGCGGCGGACGGGTGAGTAACACGTGGGCAACCTGCCCTAAAGCGGGGGATAACATT 137
L.curvatus           T-GAGTGGCGGACGGGTGAGTAACACGTGGGTAACCTGCCCTAAAGTGGGGGATAACATT 147
L.intestinalis       C-GAGCGGCGGATGGGTGAGTAACACGTGGGTAACCTGCCCTAAAGTCTGGGATACCACT 143
L.crispatus          C-GAGCGGCGGATGGGTGAGTAACACGTGGGGAACCTGCCCCATAGTCTGGGATACCACT 171
L.casei              C-GAGTGGCGGACGGGTGAGTAACACGTGGGTAACCTGCCCTTAAGTGGGGGATAACATT 145
L.kunkeei            C-GAGTGGCGAACTGGTGAGTAACACGTGGGTAACCTGCCCCGAAGCGGGGGATAACATT 151
L.zeae               C-GAGTGGCGGACGGGTGAGTAACACGTGGGTAACCTGCCCTTAAGTGGGGGATAACATT 145
L.salivarius         TTGAGTGGCGGACGGGTGAGTAACACGTGGGTAACCTGCCTAAAAGAAGGGGATAACACT 141
Bacillus-lentus      T-TAGCGGCGGACGGGTGAGTAACACGTGGGCAACCTACCTGTAAGACTGGGATAACTTC 146
                        ** **** *  ***************** ***** **    **   ****** *

Lac                  TGGAAACAGATGCTAATACCGCATAACAACTTGGA-CCGCATGGTCCGAGTTTGAAAGAT 185
L.plantarum          TGGAAACAGATGCTAATACCGCATAACAACTTGGA-CCGCATGGTCCGAGTTTGAAAGAT 198
L.panis              TGGAAACAGGTGCTAATACCGCATAACTACGAAAACC-ACATGGTTTTCGTATAAAAGAT 196
L.curvatus           TGGAAACAGATGCTAATACCGCATAAAACCTAGCACC-GCATGGTGCAAGGTTGAAAGAT 206
L.intestinalis       TGGAAACAGGTGCTAATACCGGATAACAACAATAGCT-GCATGGCTATTGCTTAAAAGGC 202
L.crispatus          TGGAAACAGGTGCTAATACCGGATAAGAACACTAGATCGCATGATCAGCTTTTAAAAGGC 231
L.casei              TGGAAACAGATGCTAATACCGCATAAATCCAAGAACC-GCATGGTTCTTGGCTGAAAGAT 204
L.kunkeei            TGGAAACAAGTGCTAATACCGCATAATTAGTTGGAACCGCATGGTTCCAACTTGAAAGAT 211
L.zeae               TGGAAACAGATGCTAATACCGCATAAATCCAAGAACC-GCATGGTTCTTGGCTGAAAGAT 204
L.salivarius         TGGAAACAGGTGCTAATACCGTATATCTCTAAGGATC-GCATGATCCTTAGATGAAAGAT 200
Bacillus-lentus      GGGAAACCGGAGCTAATACCGGATAACTTCTTTCTTC-TCCTGGAGAAAGGTTGAAAGAC 205
                      ******    ********** ***              * **         * ****

Lac                  GGCTT-CGGCTATCACTTTTGGATGGTCCCGCGGCGTATTAGCTAGATGGTGGGGTAACG 244
L.plantarum          GGCTT-CGGCTATCACTTTTGGATGGTCCCGCGGCGTATTAGCTAGATGGTGGGGTAACG 257
L.panis              GGTTT-CGGCTATCACTTTAGGATGGGCCCGCGGTGCATTAGCTAGTTGGTAGGGTAACG 255
L.curvatus           GGTTT-CGGCTATCACTTTAGGATGGACCCGCGGTGCATTAGTTAGTTGGTGAGGTAAAG 265
L.intestinalis       GGCGT-AAGCTGTCGCTAAAGGATGGACCCGCGGTGCATTAGCTAGTTGGTAAGGTAACG 261
L.crispatus          GGCGT-AAGCTGTCGCTATGGGATGGCCCCGCGGTGCATTAGCTAGTTGGTAAGGTAAAG 290
L.casei              GGCGTCAAGCTATCGCTTTTGGATGGACCCGCGGCGTATTAGCTAGTTGGTGAGGTAACG 264
L.kunkeei            GGCTC-TG-CTATCACTTTGGGATGGACCCGCGCCGTATTAGTTAGTTGGTGAGATAAAA 269
L.zeae               GGCGT-AAGCTATCGCTTTTGGATGGACCCGCGGCGTATTAGCTAGTTGGTGAGGTAACG 263
L.salivarius         GGTTC--TGCTATCGCTTTTAGATGGACCCGCGGCGTATTAACTAGTTGGTGGGGTAACG 258
Bacillus-lentus      GGCTT-CGGCTGTCACTTACAGATGGGCCCGCGGCGCATTAGCTAGTTGGTGAGGTAACG 264
                     **       ** ** **    ***** ******  * ****  *** ****  * ***
```

```
Lac                GCTCACCATGGCAATGATACGTAGCCGACCTGAGAGGGTAATCGGCCACATTGGGACTGA 304
L.plantarum        GCTCACCATGGCAATGATACGTAGCCGACCTGAGAGGGTAATCGGCCACATTGGGACTGA 317
L.panis            GCTTACCAAGGCAATGATGCATAGCCGAGTTGAGAGACTGATCGGCCACAATGGAACTGA 315
L.curvatus         GCTCACCAAGACCGTGATGCATAGCCGACCTGAGAGGGTAATCGGCCACACTGGGACTGA 325
L.intestinalis     GCTTACCAAGGCGACGATGCATAGCCGAGTTGAGAGACTGATCGGCCACATTGGGACTGA 321
L.crispatus        GCTTACCAAGGCGATGATGCATAGCCGAGTTGAGAGACTGATCGGCCACATTGGGACTGA 350
L.casei            GCTCACCAAGGCGATGATACGTAGCCGAACTGAGAGGTTGATCGGCCACATTGGGACTGA 324
L.kunkeei          GCCCACCAAGACGATGATACGTAGCCGACCTGAGAGGGTAATCGGCCACATTGGGACTGA 329
L.zeae             GCTCACCAAGGCGATGATACGTAGCCGAACTGAGAGGTTGATCGGCCACATTGGGACTGA 323
L.salivarius       GCCTACCAAGGTGATGATACGTAGCCGAACTGAGAGGTTGATCGGCCACATTGGGACTGA 318
Bacillus-lentus    GCTCACCAAGGCAACGATGCGTAGCCGACCTGAGAGGGTGATCGGCCACACTGGGACTGA 324
                   **  **** *     *** * *******  ******  * ********** *** *****

Lac                GACACGGCCCAAACTCCTACGGGAGGCAGCAGTAGGGAATCTTCCACAATGGACGAAAGT 364
L.plantarum        GACACGGCCCAAACTCCTACGGGAGGCAGCAGTAGGGAATCTTCCACAATGGACGAAAGT 377
L.panis            GACACGGTCCATACTCCTACGGGAGGCAGCAGTAGGGAATCTTCCACAATGGGCGCAAGC 375
L.curvatus         GACACGGCCCAGACTCCTACGGGAGGCAGCAGTAGGGAATCTTCCACAATGGACGAAAGT 385
L.intestinalis     GACACGGCCCAAACTCCTACGGGAGGCAGCAGTAGGGAATCTTCCACAATGGGCGAAAGC 381
L.crispatus        GACACGGCCCAAACTCCTACGGGAGGCAGCAGTAGGGAATCTTCCACAATGGACGCAAGT 410
L.casei            GACACGGCCCAAACTC-TACGGGAGGCAGCAGTAGGGAATCTTCCACAATGGACGCAAGT 383
L.kunkeei          GACACGGCCCAGACTCCTACGGGAGGCAGCAGTAGGGAATCTTCCACAATGGACGAAAGT 389
L.zeae             GACACGGCCCAAACTCCTACGGGAGGCAGCAGTAGGGAATCTTCCACAATGGACGCAAGT 383
L.salivarius       GACACGGCCCAAACTCCTACGGGAGGCAGCAGTAGGGAATCTTCCACAATGGACGCAAGT 378
Bacillus-lentus    GACACGGCCCAGACTCCTACGGGAGGCAGCAGTAGGGAATCTTCCGCAATGGACGAAAGT 384
                   ******* *** **** **************************** ****** ** ***

Lac                CTGATGGAGCAACGCCGCGTGAGTGAAGAAGGGTTTCGGCTCGTAAAACTCTGTTGTTAA 424
L.plantarum        CTGATGGAGCAACGCCGCGTGAGTGAAGAAGGGTTTCGGCTCGTAAAACTCTGTTGTTAA 437
L.panis            CTGATGGAGCAACACCGCGTGAGTGAAGAAGGGTTTCGGCTCGTAAAACTCTGTTGTTGA 435
L.curvatus         CTGATGGAGCAACGCCGCGTGAGTGAAGAAGGTTTTCGGATCGTAAAACTCTGTTGTTGG 445
L.intestinalis     CTGATGGAGCAACGCCGCGTGAGTGAAGAAGGTTTTCGGATCGTAAAGCTCTGTTGTTGG 441
L.crispatus        CTGATGGAGCAACGCCGCGTGAGTGAAGAAGGT--TCGGCTCGTAAAGCTCTGTTGGTAG 468
L.casei            CTGATGGAGCAACGCCGCGTGAGTGAAGAAGGCTTTCGGGTCGTAAAACTCTGTTGTTGG 443
L.kunkeei          CTGATGGAGCAACGCCGCGTGAGTGATGAAGGTTTTCGGATCGTAAAACTCTGTTGTTAA 449
L.zeae             CTGATGGAGCAACGCCGCGTGAGTGAAGAAGGCTTTCGGGTCGTAAAACTCTGTTGTTGG 443
L.salivarius       CTGATGGAGCAACGCCGCGTGAGTGAAGAAGGTCTTCGGATCGTAAAACTCTGTTGTTAG 438
Bacillus-lentus    CTGACGGAGCAACGCCGCGTGAGTGATGAAGGTTTTCGGATCGTAAAACTCTGTTATCAG 444
                   **** ******** ************ *****   **** ******* *******

Lac                AGAAGAACATATCTGAGAGTAACTGTTCAGGTATTGACGGTATTTAACCAGAAAGCCACG 484
L.plantarum        AGAAGAACATATCTGAGAGTAACTGTTCAGGTATTGACGGTATTTAACCAGAAAGCCACG 497
L.panis            AGAAGAACGTGCGTGAGAGTAACTGTTCATGCAGTGACGGTATTCAACCAGAAAGTCACG 495
L.curvatus         AGAAGAACGTATTTGATAGTAACTGATCAGGTAGTGACGGTATCCAACCAGAAAGCCACG 505
L.intestinalis     TGAAGAAGGATAGAGGTAGTAACTGGCCCCTATTTGACGGTAATCAACCAGAAAGTCACG 501
L.crispatus        TGAAGAAGGATAGAGGTAGTAACTGGCCTTTATTTGACGGTAATCAACCAGAAAGTCACG 528
L.casei            AGAAGAATGGTCGGCAGAGTAACTGTTGTCGGCGTGACGGTATCCAACCAGAAAGCCACG 503
L.kunkeei          AGAAGAACAAGTGTTAGAGTAACTGTTAACACTTTGACGGTATTTAACCAGAAAGCCACG 509
L.zeae             AGAAGAATGGTCGGCAGAGTAACTGTTGTCGGCGTGACGGTATCCAACCAGAAAGCCACG 503
L.salivarius       AGAAGAACACGAGTGAGAGTAACTGTTCATTCGATGACGGTATCTAACCAGCAAGTCACG 498
Bacillus-lentus    GGAAGAACAAGTATCGGAGTAACTGCCGGTACCTTGACGGTACCTGACCAGAAAGCCACG 504
                    ******          ********         ********    ***** *** ****

Lac                GCTAACTACGTGCCAGCAGCCGCGGTAATACGTAGGTGGCAAGCGTTGTCCGGATTTATT 544
L.plantarum        GCTAACTACGTGCCAGCAGCCGCGGTAATACGTAGGTGGCAAGCGTTGTCCGGATTTATT 557
L.panis            GCTAACTACGTGCCAGCAGCCGCGGTAATACGTAGGTGGCAAGCGTTATCCGGATTTATT 555
L.curvatus         GCTAACTACGTGCCAGCAGCCGCGGTAATACGTAGGTGGCAAGCGTTGTCCGGATTTATT 565
L.intestinalis     GCTAACTACGTGCCAGCAGCCGCGGTAATACGTAGGTGGCAAGCGTTGTCCGGATTTATT 561
L.crispatus        GCTAACTACGTGCCAGCAGCCGCGGTAATACGTAGGTGGCAAGCGTTGTCCGGATTTATT 588
L.casei            GCTAACTACGTGCCAGCAGCCGCGGTAATACGTAGGTGGCAAGCGTTATCCGGATTTATT 563
L.kunkeei          GCTAACTACGTGCCAGCAGCCGCGGTAATACGTAGGTGGCAAGCGTTGTCCGGATTTATT 569
L.zeae             GCTAACTACGTGCCAGCAGCCGCGGTAATACGTAGGTGGCAAGCGTTATCCGGATTTATT 563
L.salivarius       GCTAACTACGTGCCAGCAGCCGCGGTAATACGTAGGTGGCAAGCGTTGTCCGGATTTATT 558
Bacillus-lentus    GCTAACTACGTGCCAGCAGCCGCGGTAATACGTAGGTGGCAAGCGTTGTCCGGAATTATT 564
                   *********************************************** ****** *****
```

```
Lac              GGGCGTAAAGCGAGCGCAGGCGGTTTTTTAAGTCTGATGTGAAAGCCTTCGGCTCAACCG 604
L.plantarum      GGGCGTAAAGCGAGCGCAGGCGGTTTTTTAAGTCTGATGTGAAAGCCTTCGGCTCAACCG 617
L.panis          GGGCGTAAAGCGAGCGCAGGCGGTTGCTTAGGTCTGATGTGAAAGCCTTCGGCTTAACCG 615
L.curvatus       GGGCGTAAAGCGAGCGCAGGCGGTTTCTTAAGTCTGATGTGAAAGCCTTCGGCTCAACCG 625
L.intestinalis   GGGCGTAAAGCGAGCGCAGGCGGAAAGATAAGTCTGATGTGAAAGCCCCCGGCTTAACCG 621
L.crispatus      GGGCGTAAAGCGAGCGCAGGCGGTTCAATAAGTCTGATGTGAAAGCCCTCGGCTTAACCG 648
L.casei          GGGCGTAAAGCGAGCGCAGGCGGTTTTTTAAGTCTGATGTGAAAGCCCTCGGCTTAACCG 623
L.kunkeei        GGGCGTAAAGCGAGCGCAGGCGGTTTTGTAAGTCTGCTGTGAAAGCCCTCAGCTCAACTG 629
L.zeae           GGGCGTAAAGCGAGCGCAGGCGGTTTTTTAAGTCTGATGTGAAAGCCCTCGGCTTAACCG 623
L.salivarius     GGGCGTAAAGGGAACGCAGGCGGTCTTTTAAGTCTGATGTGAAAGCCTTCGGCTTAACCG 618
Bacillus-lentus  GGGCGTAAAGCGCGCGCAGGCGGTTTCTTAAGTCTGATGTGAAAGCCCACGGCTCAACCG 624
                 ********** *  *********     ** ***** **********  * *** *** *

Lac              AAGAAGTGCATCGGAAACTGGGAAACTTGAGTGCAGAAGAGGACAGTGGAACTCCATGTG 664
L.plantarum      AAGAAGTGCATCGGAAACTGGGAAACTTGAGTGCAGAAGAGGACAGTGGAACTCCATGTG 677
L.panis          AAGAAGTGCATCGGAAACCGGGCGACTTGAGTGCAGAAGAGGACAGTGGAACTCCATGTG 675
L.curvatus       AAGAAGTGCATCGGAAACTGGGAAACTTGAGTGCAGAAGAGGACAGTGGAACTCCATGTG 685
L.intestinalis   AGGAATTGCATCGGAAACTGTGTTTCTTGAGTGCAGAAGAGGAGAGTGGAACTCCATGTG 681
L.crispatus      GAGAATTGCATCAGAAACTGTTGAACTTGAGTGCAGAAGAGGAGAGTGGAACTCCATGTG 708
L.casei          AGGAAGCGCATCGGAAACTGGGAAACTTGAGTGCAGAAGAGGACAGTGGAACTCCATGTG 683
L.kunkeei        AGGAAGTGCAGTGGAAACTACAAAACTTGAGTACAGAAGAGGAAAGTGGAACTCCATGTG 689
L.zeae           AGGAAGCGCATCGGAAACTGGGAAACTTGAGTGCAGAAGAGGACAGTGGAACTCCATGTG 683
L.salivarius     GAGTAGTGCATTGGAAACTGGAAGACTTGAGTGCAGAAGAGGAGAGTGGAACTCCATGTG 678
Bacillus-lentus  TGGAAGGTCATTGGAAACTGGGAGACTTGAGTGCAGAAGAGAAGAGCGGAATTCCACGTG 684
                   * *   **   *****       ******* ******** * ** **** **** ***

Lac              TAGCGGTGAAATGCGTAGATATATGGAAGAACACCAGTGGCGAAGGCGGCTGTCTGGTCT 724
L.plantarum      TAGCGGTGAAATGCGTAGATATATGGAAGAACACCAGTGGCGAAGGCGGCTGTCTGGTCT 737
L.panis          TAGCGGTGGAATGCGTAGATATATGGAAGAACACCAGTGGCGAAGGCGGCTGTCTGGTCT 735
L.curvatus       TAGCGGTGAAATGCGTAGATATATGGAAGAACACCAGTGGCGAAGGCGGCTGTCTGGTCT 745
L.intestinalis   TAGCGGTGGAATGCGTAGATATATGGAAGAACACCAGTGGCGAAGGCGGCTCTCTGGTCT 741
L.crispatus      TAGCGGTGGAATGCGTAGATATATGGAAGAACACCAGTGGCGAAGGCGGCTCTCTGGTCT 768
L.casei          TAGCGGTGAAATGCGTAGATATATGGAAGAACACCAGTGGCGAAGGCGGCTGTCTGGTCT 743
L.kunkeei        TAGCGGTGAAATGCGTAGATATATGGAAGAACACCAGTGGCGAAGGCGGCTTTCTGGTCT 749
L.zeae           TAGCGGTGAAATGCGTAGATATATGGAAGAACACCAGTGGCGAAGGCGGCTGTCTGGTCT 743
L.salivarius     TAGCGGTGAAATGCGTAGATATATGGAAGAACACCAGTGGCGAAAGCGGCTCTCTGGTCT 738
Bacillus-lentus  TAGCGGTGAAATGCGTAGAGATGTGGAGGAACACCAGTGGCGAAGGCGGCTCTTTGGTCT 744
                 ******** ********** ** **** *************** ****** * ******

Lac              GTAACTGACGCTGAGGCTCGAAAGTATGGGTAGCAAACAGGATTAGATACCCTGGTAGTC 784
L.plantarum      GTAACTGACGCTGAGGCTCGAAAGTATGGGTAGCAAACAGGATTAGATACCCTGGTAGTC 797
L.panis          GCAACTGACGCTGAGGCTCGAAAGCATGGGTAGCGAACAGGATTAGATACCCTGGTAGTC 795
L.curvatus       GTAACTGACGCTGAGGCTCGAAAGCATGGGTAGCAAACAGGATTAGATACCCTGGTAGTC 805
L.intestinalis   GTAACTGACGCTGAGGCTCGAAAGCATGGGTAGCGAACAGGATTAGATACCCTGGTAGTC 801
L.crispatus      GCAACTGACGCTGAGGCTCGAAAGCATGGGTAGCGAACAGGATTAGATACCCTGGTAGTC 828
L.casei          GTAACTGACGCTGAGGCTCGAAAGCATGGGTAGCGAACAGGATTAGATACCCTGGTAGTC 803
L.kunkeei        GTTACTGACGCTGAGGCTCGAAAGCATGGGTAGCGAACAGGATTAGATACCCTGGTAGTC 809
L.zeae           GTAACTGACGCTGAGGCTCGAAAGCATGGGTAGCGAACAGGATTAGATACCCTGGTAGTC 803
L.salivarius     GTAACTGACGCTGAGGTTCGAAAGCGTGGGTAGCAAACAGGATTAGATACCCTGGTAGTC 798
Bacillus-lentus  GTAACTGACGCTGAGGCGCGAAAGCGTGGGGAGCGAACAGGATTAGATACCCTGGTAGTC 804
                 *  *************  ******  **** *** ************************

Lac              CATACCGTAAACGATGAATGCTAAGTGTTGGAGGGTTTCCGCCCTTCAGTGCTGCAGCTA 844
L.plantarum      CATACCGTAAACGATGAATGCTAAGTGTTGGAGGGTTTCCGCCCTTCAGTGCTGCAGCTA 857
L.panis          CATGCCGTAAACGATGAGTGCTAGGTGTTGGAGGGTTTCCGCCCTTCAGTGCCGAAGCTA 855
L.curvatus       CATGCCGTAAACGATGAGTGCTAGGTGTTGGAGGGTTTCCGCCCTTCAGTGCCGCAGCTA 865
L.intestinalis   CATGCCGTAAACGATGAGTGCTAAGTGTTGGGAGGTTTCCGCCTCTCAGTGCTGCAGCTA 861
L.crispatus      CATGCCGTAAACGATGAGTGCTAAGTGTTGGGAGGTTTCCGCCTCTCAGTGCTGCAGCTA 888
L.casei          CATGCCGTAAACGATGAATGCTAGGTGTTGGAGGGTTTCCGCCCTTCAGTGCCGCAGCTA 863
L.kunkeei        CATGCCGTAAACGATGAATACTAGGTGTTGGAGGGTTTCCGCCCTTCAGTGCCGCAGCTA 869
L.zeae           CATGCCGTAAACGATGAATGCTAGGTGTTGGAGGGTTTCCGCCCTTCAGTGCCGCAGCTA 863
L.salivarius     CACGCCGTAAACGATGAATGCTAGGTGTTGGAGGGTTTCCGCCCTTCAGTGCCGCAGCTA 858
Bacillus-lentus  CACGCCGTAAACGATGAGTGCTAAGTGTTAGAGGGTTTCCGCCCTTTAGTGCTGCAGTTA 864
                 **  ************* * *** ***** *  **********  * ***** * ** **
```

```
Lac                 ACGCATTAAGCATTCCGCCTGGGGAGTACGGCCGCAAGGCTGAAACTCAAAGGAATTGAC 904
L.plantarum         ACGCATTAAGCATTCCGCCTGGGGAGTACGGCCGCAAGGCTGAAACTCAAAGGAATTGAC 917
L.panis             ACGCATTAAGCACTCCGCCTGGGGAGTACGACCGCAAGGTTGAAACTCAAAGGAATTGAC 915
L.curvatus          ACGCATTAAGCACTCCGCCTGGGGAGTACGACCGCAAGGTTGAAACTCAAAGGAATTGAC 925
L.intestinalis      ACGCATTAAGCACTCCGCCTGGGGAGTACGACCGCAAGGTTGAAACTCAAAGGAATTGAC 921
L.crispatus         ACGCATTAAGCACTCCGCCTGGGGAGTACGACCGCAAGGTTGAAACTCAAAGGAATTGAC 948
L.casei             ACGCATTAAGCATTCCGCCTGGGGAGTACGACCGCAAGGTTGAAACTCAAAGGAATTGAC 923
L.kunkeei           ACGCATTAAGTATTCCGCCTGGGGAGTACGACCGCAAGGTTGAAACTCAAAGGAATTGAC 929
L.zeae              ACGCATTAAGCATTCCGCCTGGGGAGTACGACCGCAAGGTTGAAACTCAAAGGAATTGAC 923
L.salivarius        ACGCAATAAGCATTCCGCCTGGGGAGTACGACCGCAAGGTTGAAACTCAAAGGAATTGAC 918
Bacillus-lentus     ACGCATTAAGCACTCCGCCTGGGGAGTACGGCCGCAAGGCTGAAACTCAAAGGAATTGAC 924
                    ***** **** * **************** ******** *******************

Lac                 GGGGGCCCGCACAAGCGGTGGAGCATGTGGTTTAATTCGAAGCTACGCGAAGAACCTTAC 964
L.plantarum         GGGGGCCCGCACAAGCGGTGGAGCATGTGGTTTAATTCGAAGCTACGCGAAGAACCTTAC 977
L.panis             GGGGGCCCGCACAAGCGGTGGAGCATGTGGTTTAATTCGAAGCTACGCGAAGAACCTTAC 975
L.curvatus          GGGGGCCCGCACAAGCGGTGGAGCATGTGGTTTAATTCGAAGCAACGCGAAGAACCTTAC 985
L.intestinalis      GGGGGCCCGCACAAGCGGTGGAGCATGTGGTTTAATTCGAAGCAACGCGAAGAACCTTAC 981
L.crispatus         GGGGGCCCGCACAAGCGGTGGAGCATGTGGTTTAATTCGAAGCAACGCGAAGAACCTTAC 1008
L.casei             GGGGGCCCGCACAAGCGGTGGAGCATGTGGTTTAATTCGAAGCAACGCGAAGAACCTTAC 983
L.kunkeei           GGGGGCCCGCACAAGTGGTGGAGCATGTGGTTTAATTCGATGCTACGCGAAGAACCTTAC 989
L.zeae              GGGGGCCCGCACAAGCGGTGGAGCATGTGGTTTAATTCGAAGCAACGCGAAGAACCTTAC 983
L.salivarius        GGGGGCCCGCACAAGCGGTGGAGCATGTGGTTTAATTCGAAGCAACGCGAAGAACCTTAC 978
Bacillus-lentus     GGGGGCCCGCACAAGCGGTGGAGCATGTGGTTTAATTCGAAGCAACGCGAAGAACCTTAC 984
                    *************** *********************** ** ***************

Lac                 CAGGTCTTGACATACTATGCAAATCTAAGAGATTAGA-CGTTCCCTTCGGGG-ACATGGA 1022
L.plantarum         CAGGTCTTGACATACTATGCAAATCTAAGAGATTAGA-CGTTCCCTTCGGGG-ACATGGA 1035
L.panis             CAGGTCTTGACATCTTGCGCTAACCTAAGAGATTAGG-CGTTCCCTTCGGGG-ACG-CAA 1032
L.curvatus          CAGGTCTTGACATCCTTTGACCACTCTAGAGATAGAG-CTTTCCCTTCGGGG-ACA-AAG 1042
L.intestinalis      CAGGTCTTGACATCTAGTGCCATCCTAAGAGATTAGG-AGTTCCCTTCGGGG-ACG-CTA 1038
L.crispatus         CAGGTCTTGACATCTAGTGCCATTTGTAGAGATACAA-AGTTCCCTTCGGGG-ACG-CTA 1065
L.casei             CAGGTCTTGACATCTTTTGATCACCTGAGAGATCAGG-TTTCCCCTTCGGGG-GCA-AAA 1040
L.kunkeei           CAGCTCTTGACATCTTCTGCCAACCCAAGAGATTGGG-CGTTCCCTTCGGGG-ACA-GAA 1046
L.zeae              CAGGTCTTGACATCTTTTGATCACCTGAGAGATCAGG-TTTCCCCTTCGGGG-GCA-AAA 1040
L.salivarius        CAGGTCTTGACATCCTTTGACCACCTAAGAGATTAGG-CTTTCCCTTCGGGG-ACA-AAG 1035
Bacillus-lentus     CAGGTCTTGACATCCTCTGACCACCCTAGAGATAGGGACTTCCCCTTCGGGGGACA-GAG 1043
                    *** *********     *        ******       * *********  *

Lac                 T-ACAGGTGGTGCATGGTTGTCGTCAGCTCGTGTCGTGAGATGTTGGGTTAAGTCCCGCA 1081
L.plantarum         T-ACAGGTGGTGCATGGTTGTCGTCAGCTCGTGTCGTGAGATGTTGGGTTAAGTCCCGCA 1094
L.panis             TGACAGGTGGTGCATGGTCGTCGTCAGCTCGTGTCGTGAGATGTTGGGTTAAGTCCCGCA 1092
L.curvatus          TGACAGGTGGTGCATGGTTGTCGTCAGCTCGTGTCGTGAGATGTTGGGTTAAGTCCCGCA 1102
L.intestinalis      AGACAGGTGGTGCATGGCTGTCGTCAGCTCGTGTCGTGAGATGTTGGGTTAAGTCCCGCA 1098
L.crispatus         AGACAGGTGGTGCATGGCTGTCGTCAGCTCGTGTCGTGAGATGTTGGGTTAAGTCCCGCA 1125
L.casei             TGACAGGTGGTGCATGGTTGTCGTCAGCTCGTGTCGTGAGATGTTGGGTTAAGTCCCGCA 1100
L.kunkeei           TGACAGGTGGTGCATGGTTGTCGTCAGCTCGTGTCGTGAGATGTTGGGTTAAGTCCCGCA 1106
L.zeae              TGACAGGTGGTGCATGGTTGTCGTCAGCTCGTGTCGTGAGATGTTGGGTTAAGTCCCGCA 1100
L.salivarius        TGACAGGTGGTGCATGGCTGTCGTCAGCTCGTGTCGTGAGATGTTGGGTTAAGTCCCGCA 1095
Bacillus-lentus     TGACAGGTGGTGCATGGTTGTCGTCAGCTCGTGTCGTGAGATGTTGGGTTAAGTCCCGCA 1103
                      *************** ****************************************

Lac                 ACGAGCGCAACCCTTATTATCAGTTGCCAGCATTAAGTTGGGCACTCTGGTGAGACTGCC 1141
L.plantarum         ACGAGCGCAACCCTTATTATCAGTTGCCAGCATTAAGTTGGGCACTCTGGTGAGACTGCC 1154
L.panis             ACGAGCGCAACCCTTGTTACTAGTTGCCAGCATTCAGTTGGGCACTCTAGTGAGACTGCC 1152
L.curvatus          ACGAGCGCAACCCTTATTACTAGTTGCCAGCATTTAGTTGGGCACTCTAGTGAGACTGCC 1162
L.intestinalis      ACGAGCGCAACCCTTGTTATTAGTTGCCAGCATTAAGTTGGGCACTCTAATGAGACTGCC 1158
L.crispatus         ACGAGCGCAACCCTTGTTATTAGTTGCCAGCATTAAGTTGGGCACTCTAATGAGACTGCC 1185
L.casei             ACGAGCGCAACCCTTATGACTAGTTGCCAGCATTGAGTTGGGCACTCTAGTAAGACTGCC 1160
L.kunkeei           ACGAGCGCAACCCTTATTATTAGTTGCCAGCATTTAGTTGGGCACTCTAGTGAGACTGCC 1166
L.zeae              ACGAGCGCAACCCTTATGACTAGTTGCCAGCATTYAGTTGGGCACTCTAGTAAGACTGCC 1160
L.salivarius        ACGAGCGCAACCCTTGTTGTCAGTTGCCAGCATTAAGTTGGGCACTCTGGCGAGACTGCC 1155
Bacillus-lentus     ACGAGCGCAACCCTTAACCTTAGTTGCCAGCATTCAGTTGGGCACTCTAAGGTGACTGCC 1163
                    ***************      ************* *************     *******
```

```
Lac                 GGTGACAAACCGGAGGAAGGTGGGGATGACGTCAAATCATCATGCCCCTTATGACCTGGG 1201
L.plantarum         GGTGACAAACCGGAGGAAGGTGGGGATGACGTCAAATCATCATGCCCCTTATGACCTGGG 1214
L.panis             GGTGACAAACCGGAGGAAGGTGGGGACGACGTCAGATCATCATGCCCCTTATGACCTGGG 1212
L.curvatus          GGTGACAAACCGGAGGAAGGTGGGGACGACGTCAAATCATCATGCCCCTTATGACCTGGG 1222
L.intestinalis      GGTGACAAACCGGAGGAAGGTGGGGATGACGTCAAGTCATCATGCCCCTTATGACCTGGG 1218
L.crispatus         GGTGACAAACCGGAGGAAGGTGGGGATGACGTCAAGTCATCATGCCCCTTATGACCTGGG 1245
L.casei             GGTGACAAACCGGAGGAAGGTGGGGATGACGTCAAATCATCATGCCCCTTATGACCTGGG 1220
L.kunkeei           GGTGATAAACCGGAGGAAGGTGGGGACGACGTCAAATCATCATGCCCCTTATGAGCTGGG 1226
L.zeae              GGTGACAAACCGGAGGAAGGTGGGGATGACGTCAAATCATCATGCCCCTTATGACCTGGG 1220
L.salivarius        GGTGACAAACCGGAGGAAGGTGGGGACGACGTCAAGTCATCATGCCCCTTATGACCTGGG 1215
Bacillus-lentus     GGTGACAAACCGGAGGAAGGTGGGGATGACGTCAAATCATCATGCCCCTTATGACCTGGG 1223
                    ***** ******************** *******  ****************** *****

Lac                 CTACACACGTGCTACAATGGATGGTACAACGAGTTGCGAACTCGCGAGAGTAAGCTAATC 1261
L.plantarum         CTACACACGTGCTACAATGGATGGTACAACGAGTTGCGAACTCGCGAGAGTAAGCTAATC 1274
L.panis             CTACACACGTGCTACAATGGCCGGTACAACGAGCAGCTAACCCGCGAGGGTGTGCAAATC 1272
L.curvatus          CTACACACGTGCTACAATGGATGGTACAAC------------------------------ 1252
L.intestinalis      CTACACACGTGCTACAATGGGCAGTACAACGAGAAGCGAGCCTGCGAAGGCAAGCGGATC 1278
L.crispatus         CTACACACGTGCTACAATGGGCAGTACAACGAGAAGCGAGCCTGCGAAGGCAAGCGAATC 1305
L.casei             CTACACACGTGCTACAATGGATGGTACAACGAGTTGCGAGACCGCGAGGTCAAGCTAATC 1280
L.kunkeei           CTACACACGTGCTACAATGGATGGTACAACGAGTCGCGAAACCGCGAGGTCAAGCTAATC 1286
L.zeae              CTACACACGTGCTACAATGGATGGTACAACGAGTTGCGAGACCGCGAGGTCAAGCTAATC 1280
L.salivarius        CTACACACGTGCTACAATGGACGGTACAACGAGTCGCGAGACCGCGAGGTTTAGCTAATC 1275
Bacillus-lentus     CTACACACGTGCTACAATGGATGGTACAAAGGGTTGCAAGACCGCGAGGTTTAGCTAATC 1283
                    ********************   ******

Lac                 TCTTAAAGCCATTCTCAGTTCGGATTGTAGGCTGCAACTCGCCTACATGAAGTCGGAATC 1321
L.plantarum         TCTTAAAGCCATTCTCAGTTCGGATTGTAGGCTGCAACTCGCCTACATGAAGTCGGAATC 1334
L.panis             TCTTAAAGCCGGTCTCAGTTCGGACTGCAGTCTGCAACTCGACTGCACGAAGTCGGAATC 1332
L.curvatus          ------------------------------------------------------------
L.intestinalis      TCTTAAAGCTGTTCTCAGTTCGGACTGCAGTCTGCAACTCGACTGTACGAAGCTGGAATC 1338
L.crispatus         TCTGAAAGCTGTTCTCAGTTCGGACTGCAGTCTGCAACTCGACTGCACGAAGCTGGAATC 1365
L.casei             TCTTAAAGCCATTCTCAGTTCGGACTGTAGGCTGCAACTCGCCTACACGAAGTCGGAATC 1340
L.kunkeei           TCTTAAAGCCATTCTCAGTTCGGATTGCAGGCTGCAACTCGCCTGCATGAAGTTGGAATC 1346
L.zeae              TCTTAAAGCCATTCTCAGTTCGGACTGTAGGCTGCAACTCGCCTACACGAAGTCGGAATC 1340
L.salivarius        TCTTAAAGCCGTTCTCAGTTCGGATTGTAGGCTGCAACTCGCCTACATGAAGTCGGAATC 1335
Bacillus-lentus     CCATAAAACCATTCTCAGTTCGGATTGCAGGCTGCAACTCGCCTGCATGAAGCCGGAATC 1343


Lac                 GCTAGTAATCGCGGATCAGCATGCCGCGGTGAATACGTTCCCGGGCCTTGTACACACCGC 1381
L.plantarum         GCTAGTAATCGCGGATCAGCATGCCGCGGTGAATACGTTCCCGGGCCTTGTACACACCGC 1394
L.panis             GCTAGTAATCGCGGATCAGCATGCCGCGGTGAATACGTTCCCGGGCCTTGTACACACCGC 1392
L.curvatus          ------------------------------------------------------------
L.intestinalis      GCTAGTAATCGCGGATCAGCACGCCGCGGTGAATACGTTCCCGGGCCTTGTACACACCGC 1398
L.crispatus         GCTAGTAATCGCGGATCAGCACGCCGCGGTGAATACGTTCCCGGGCCTTGTACACACCGC 1425
L.casei             GCTAGTAATCGCGGATCAGCACGCCGCGGTGAATACGTTCCCGGGCCTTGTACACACCGC 1400
L.kunkeei           ACTAGTAATCGTGGATCAGCATGCCACGGTGAATACGTTCCCGGGCCTTGTACACACCGC 1406
L.zeae              GCTAGTAATCGCGGATCAGCACGCCGCGGTGAATACGTTCCCGGGCCTTGTACACACCGC 1400
L.salivarius        GCTAGTAATCGCGAATCAGCATGTCGCGGTGAATACGTTCCCGGGCCTTGTACACACCGC 1395
Bacillus-lentus     GCTAGTAATCGTGGATCAGCATGCCACGGTGAATACGTTCCCGGGCCTTGTACACACCGC 1403


Lac                 CCGTCACACCATGAGAGTTTGTAACACCCAAAGTCGGTGGGGTAACCTTTTA--GGAACC 1439
L.plantarum         CCGTCACACCATGAGAGTTTGTAACACCCAAAGTCGGTGGGGTAACCTTTTA--GGAACC 1452
L.panis             CCGTCACACCATGGAAGTTTGCAATGCCCAAAGTCAGTGGCCTAACCATTTG---GAGGG 1449
L.curvatus          ------------------------------------------------------------
L.intestinalis      CCGTCACACCATGGAAGTCTGCAATGCCCGAAGCCGGTGGCCTAACCACTTATGTGGAAG 1458
L.crispatus         CCGTCACACCATGGGAGTCTGCAATGCCCAAAGCCGGTGGGATAACC-TTTATAGGAGTC 1484
L.casei             CCGTCACACCATGAGAGTTTGTAACACCCGAAGCCGGTGGCGTAACCCTTTTAGGGAGCG 1460
L.kunkeei           CCGTCACACCATGAGAGTTTGTAACACCCAAAGACGATGGGGTAACCTTTTA--GGAGCT 1464
L.zeae              CCGTCACACCATGAGAGTTTGTAACACCCGAAGCCGGTGGCGTAACCCTTTTAGGGAGCG 1460
L.salivarius        CCGTCACACCATGAGAGTTTGTAACACCCAAAGCCGGTGGGGTAACCGC---AAGGAGCC 1452
Bacillus-lentus     CCGTCACACCACGAGAGTTTGTAACACCCGAAGTCGGTGGGGTAACC-CTTACGGGAGCC 1462
```

```
Lac                  AGCCGCCTAAG------------------------------------------------- 1450
L.plantarum          AGCCGCCTAAGG------------------------------------------------ 1464
L.panis              AGCTGCCTAAGGCAGGACAGATGACTGGGGTGAAGTCGTAA------------------- 1490
L.curvatus           ------------------------------------------------------------
L.intestinalis       AGCCGTCTAAGGCAGGGCAGATGACTGGGGTGAAGTCGTAACAAGGTAGCCGTAG----- 1513
L.crispatus          AGCCGTCTAAGGTAGGACAGATGATTAGGGTGAAGTCGTGACAAGGTAGCCGTAG----- 1539
L.casei              AGCCGTCTAAGGTGGGACAAATGATTAGGGTGAAGTCGTAACAAGGTAGCCGTAGGAGAA 1520
L.kunkeei            AGTCGTCTAAGGTGGGACAGATGATTAGGGTGAAGTCGTAACAAGGTAGCCGT------- 1517
L.zeae               AGCCGTCTAAGGTGGGACAAATGATTAGGGTGAAGTCGTAACAAGGTAGCCGTAGGAGAA 1520
L.salivarius         AGCCGTCTAAGGTGGGACAGATGATTGGGGTGAAGTCGTAACAAGGTAGCCGTAGGAGAA 1512
Bacillus-lentus      AGCCGCCGAAGGTGGGACAGATGATTGGGGTGAAGTCGTAACAAGGTAGC-GTATCGGAA 1521

Lac                  --------------
L.plantarum          --------------
L.panis              --------------
L.curvatus           --------------
L.intestinalis       --------------
L.crispatus          --------------
L.casei              CC------------ 1522
L.kunkeei            --------------
L.zeae               CC------------ 1522
L.salivarius         CCTGC--------- 1517
Bacillus-lentus      GGTGCGGTGGATCA 1535
```

Phylogenetic Tree

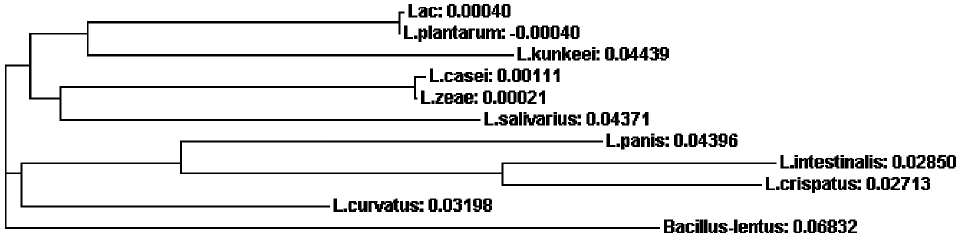

Lineage:

Root; domain Bacteria; phylum Firmicutes; class Bacilli; order Lactobacillales; family Lactobacillaceae; genus Lactobacillus

Bacillus lentus (outgroup: AB021189); *Lactobacillus curvatus* (AJ270951); *Lactobacillus crispatus* (AJ242969); *Lactobacillus intestinalis* (AJ306299); *Lactobacillus panis* (X94230); *Lactobacillus salivarius* (AF335475); *Lactobacillus zeae* (D86516); *Lactobacillus casei* (D16551); *Lactobacillus kunkeei* (Y11374); *Lactobacillus plantarum* (JQ340030); Lac (query sequence).
