# Supplementary material for: Biotransformation of Momordica charantia fresh juice by Lactobacillus plantarum BET003 and its putative anti-diabetic potential
Source: PeerJ. 2015 Oct 29;3:e1376. doi: 10.7717/peerj.1376 (PMC4631465; doi:10.7717/peerj.1376)
Supplement: Supplemental Information 3 [file peerj-03-1376-s003.docx]

**Manuscript ID: #2015:06:5357**

Data for ANOVA (not previously shown)

|  |  |  |  |  |
| --- | --- | --- | --- | --- |
| 7 | 1 | 0.00 | -1.41 | 0.57 |
| 10 | 2 | 0.00 | 0.00 | 0.59 |
| 3 | 3 | -1.00 | 1.00 | 0.3 |
| 4 | 4 | 1.00 | 1.00 | 0.59 |
| 2 | 5 | 1.00 | -1.00 | 0.64 |
| 1 | 6 | -1.00 | -1.00 | 0.24 |
| 12 | 7 | 0.00 | 0.00 | 0.64 |
| 8 | 8 | 0.00 | 1.41 | 0.58 |
| 11 | 9 | 0.00 | 0.00 | 0.51 |
| 5 | 10 | -1.41 | 0.00 | 0.36 |
| 6 | 11 | 1.41 | 0.00 | 0.55 |
| 13 | 12 | 0.00 | 0.00 | 0.59 |
| 9 | 13 | 0.00 | 0.00 | 0.64 |

**Response** **1** **specific growth rate** **Transform:** **None**
  ***** WARNING: The Cubic Model and higher are Aliased! *****

  **Summary (detailed tables shown below)**
 **Sequential** **Lack of Fit** **Adjusted** **Predicted**
 **Source** **p-value** **p-value** **R-Squared** **R-Squared**

Linear 0.0232 0.0631 0.4349 0.1341 Suggested
 2FI 0.6132 0.0505 0.3907 -0.4117
 Quadratic 0.0580 0.1128 0.6527 -0.1517 Suggested
 Cubic 0.1732 0.1262 0.7588 -2.1779 Aliased

  **Sequential Model Sum of Squares [Type I]**

**Sum of** **Mean** **F** **p-value**
 **Source** **Squares** **df** **Square** **Value** **Prob > F**
 Mean vs Total 3.56 1 3.56

Linear vs Mean 0.11 2 0.057 5.62 0.0232 Suggested
 2FI vs Linear 3.025E-003 1 3.025E-003 0.27 0.6132
 Quadratic vs 2FI 0.055 2 0.028 4.39 0.0580 Suggested
 Cubic vs Quadratic 0.022 2 0.011 2.54 0.1732 Aliased
 Residual 0.022 5 4.367E-003
 Total 3.77 13 0.29

 *"Sequential Model Sum of Squares [Type I]"*: Select the highest order polynomial where the
 additional terms are significant and the model is not aliased.

**Lack of Fit Tests**
 **Sum of** **Mean** **F** **p-value**
 **Source** **Squares** **df** **Square** **Value** **Prob > F**
 Linear 0.091 6 0.015 5.36 0.0631 Suggested
 2FI 0.088 5 0.018 6.22 0.0505
 Quadratic 0.033 3 0.011 3.85 0.1128 Suggested
 Cubic 0.011 1 0.011 3.71 0.1262 Aliased
 Pure Error 0.011 4 2.830E-003

 *"Lack of Fit Tests"*: Want the selected model to have insignificant lack-of-fit.


  **Model Summary Statistics**
 **Std.** **Adjusted** **Predicted**
 **Source** **Dev.** **R-Squared** **R-Squared** **R-Squared** **PRESS**

Linear 0.10 0.5291 0.4349 0.1341 0.19 Suggested
 2FI 0.11 0.5430 0.3907 -0.4117 0.31
 Quadratic 0.079 0.7974 0.6527 -0.1517 0.25 Suggested
 Cubic 0.066 0.8995 0.7588 -2.1779 0.69 Aliased

 *"Model Summary Statistics"*: Focus on the model maximizing the "Adjusted R-Squared"
 and the "Predicted R-Squared".

Use your mouse to right click on individual cells for definitions.
 **Response** **1** **specific growth rate**
  **ANOVA for Response Surface Quadratic Model**
 **Analysis of variance table [Partial sum of squares - Type III]**
 **Sum of** **Mean** **F** **p-value**
 **Source** **Squares** **df** **Square** **Value** **Prob > F**

Model 0.17 5 0.035 5.51 0.0225significant

*A-RPM* *0.11* *1* *0.11* *18.27* *0.0037*
  *B-Initial vol.* *7.286E-005* *1* *7.286E-005* *0.012* *0.9173*
  *AB* *3. 025E-003* *1* *3.025E-003* *0.48* *0.5103*
  *A2* *0.053* *1* *0.053* *8.49* *0.0225*
  *B2* *5.309E-003* *1* *5.309E-003* *0.84* *0.3888*
 Residual 0.044 7 6.289E-003
 *Lack of Fit* *0.033* *3* *0.011* *3.85* *0.1128not significant*
 *Pure Error* *0.011* *4* *2.830E-003*
 Cor Total 0.22 12

 The Model F-value of 5.51 implies the model is significant. There is only
 a 2.25% chance that a "Model F-Value" this large could occur due to noise.

 Values of "Prob > F" less than 0.0500 indicate model terms are significant.
 In this case A, A2 are significant model terms.
 Values greater than 0.1000 indicate the model terms are not significant.
 If there are many insignificant model terms (not counting those required to support hierarchy),
 model reduction may improve your model.

 The "Lack of Fit F-value" of 3.85 implies the Lack of Fit is not significant relative to the pure
 error. There is a 11.28% chance that a "Lack of Fit F-value" this large could occur due
 to noise. Non-significant lack of fit is good -- we want the model to fit.

Std. Dev. 0.079 R-Squared 0.7974
 Mean 0.52 Adj R-Squared 0.6527
 C.V. % 15.16 Pred R-Squared -0.1517

PRESS 0.25 Adeq Precision 6.938

 A negative "Pred R-Squared" implies that the overall mean is a better predictor of your
 response than the current model.

 "Adeq Precision" measures the signal to noise ratio. A ratio greater than 4 is desirable. Your
 ratio of 6.938 indicates an adequate signal. This model can be used to navigate the design space.

**Coefficient** **Standard** **95% CI** **95% CI**

**Factor** **Estimate** **df** **Error** **Low** **High** **VIF**

Intercept 0.59 1 0.035 0.51 0.68

A-RPM 0.12 1 0.028 0.054 0.19 1.00
 B-Initial vol. 3.018E-003 1 0.028 -0.063 0.069 1.00
 AB -0.028 1 0.040 -0.12 0.066 1.00
 A2 -0.088 1 0.030 -0.16 -0.017 1.02
 B2 -0.028 1 0.030 -0.099 0.043 1.02

**Final Equation in Terms of Coded Factors:**

 specific growth rate =
 +0.59
 +0.12 * A
 +3.018E-003 * B
 -0.028 * A * B
 -0.088 * A2
 -0.028 * B2

**Final Equation in Terms of Actual Factors:**

 specific growth rate =
 +0.59400
 +0.11984 * RPM
 +3.01777E-003 * Initial vol.
 -0.027500 * RPM * Initial vol.
 -0.087625 * RPM2
 -0.027625 * Initial vol.2

The Diagnostics Case Statistics Report has been moved to the Diagnostics Node.
 In the Diagnostics Node, Select Case Statistics from the View Menu.

 Proceed to Diagnostic Plots (the next icon in progression). Be sure to look at the:
 1) Normal probability plot of the studentized residuals to check for normality of residuals.
 2) Studentized residuals versus predicted values to check for constant error.
 3) Externally Studentized Residuals to look for outliers, i.e., influential values.
 4) Box-Cox plot for power transformations.

 If all the model statistics and diagnostic plots are OK, finish up with the Model Graphs icon.

**Factor** **Name** **Level** **Low Level** **High Level** **Std. Dev.** **Coding**

**A** **RPM** **0.73** **-1.00** **1.00** **0.000** **Actual**

**B** **Initial vol.** **-0.31** **-1.00** **1.00** **0.000** **Actual**

**99% of** **Population**
 **Response** **Prediction** **Std Dev** **SE Mean** **95% CI low** **95% CI high** **SE Pred** **95% PI low** **95% PI high** **95% TI low** **95% TI high**
 specific growth rate 0.63742 0.0793017 0.0363463 0.551474 0.723365 0.0872342 0.431143 0.843696 0.201459 1.07338

**Confirmation Report**
 **Two-sided** **Confidence =**  **95%** **n =**  **1**

**Factor** **Name** **Level** **Low Level** **High Level** **Std. Dev.** **Coding**

**A** **RPM** **0.73** **-1.00** **1.00** **0.000** **Actual**

**B** **Initial vol.** **-0.31** **-1.00** **1.00** **0.000** **Actual**

**Response** **Prediction** **Std Dev** **SE (n=1)** **95% PI low** **95% PI high**
 specific growth rate 0.63742 0.0793017 0.0872369 0.431137 0.843702
