# Supplementary figures and images for: Biotransformation of Momordica charantia fresh juice by Lactobacillus plantarum BET003 and its putative anti-diabetic potential
Source: PeerJ. 2015 Oct 29;3:e1376. doi: 10.7717/peerj.1376 (PMC4631465; doi:10.7717/peerj.1376)

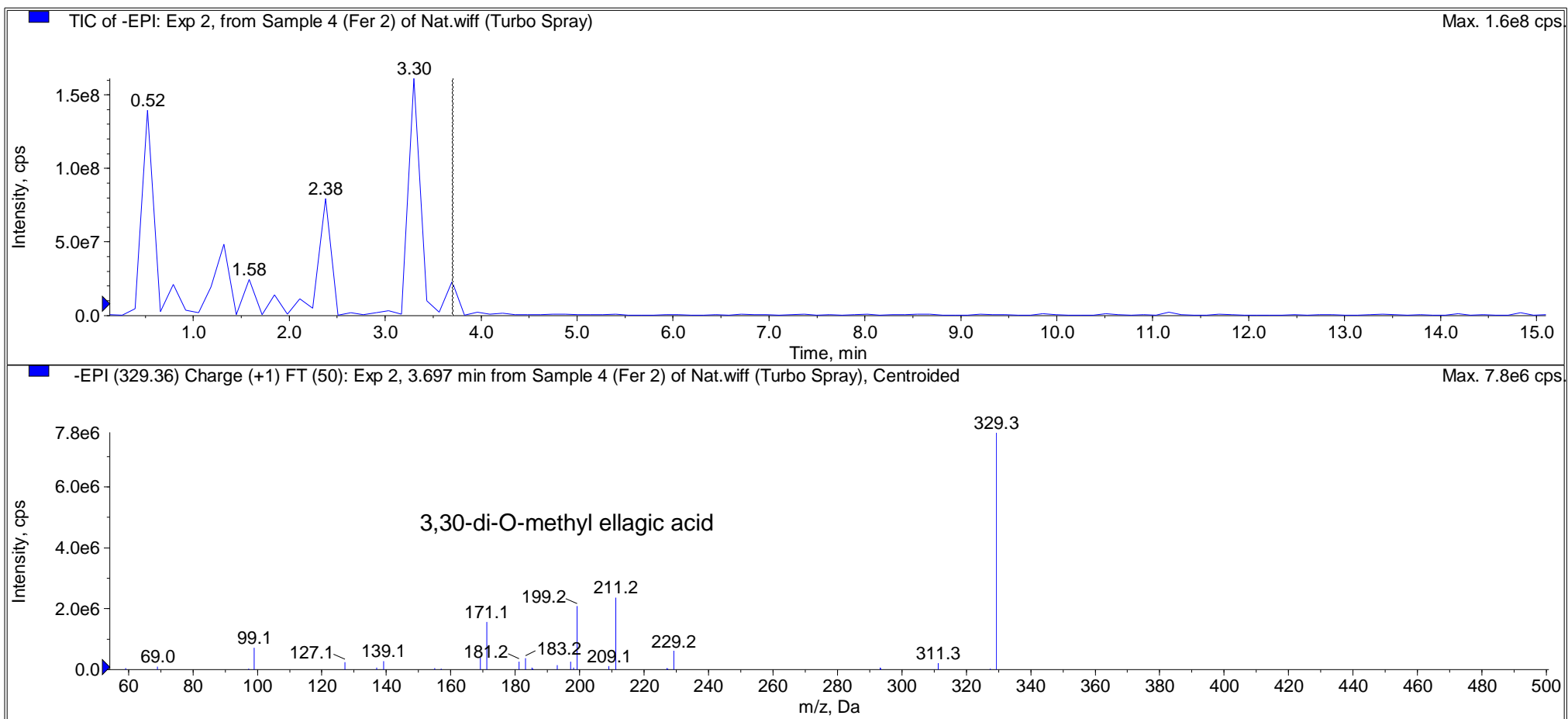

Supplement: Supplemental Information 4 [file peerj-03-1376-s004.pdf]

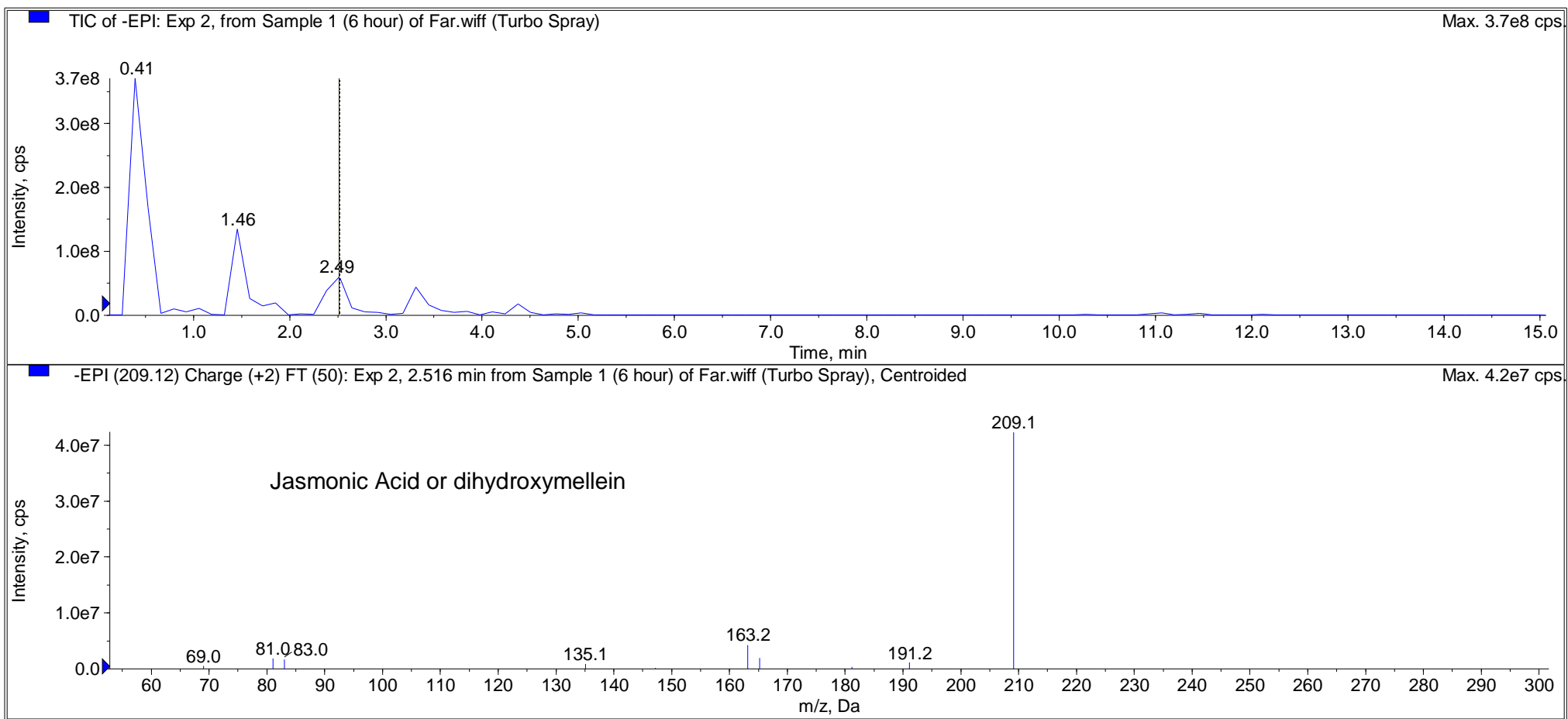

Supplement: Supplemental Information 5 [file peerj-03-1376-s005.pdf]
